# Surface PEGylation suppresses pulmonary effects of CuO in allergen-induced lung inflammation

**DOI:** 10.1186/s12989-019-0309-1

**Published:** 2019-07-05

**Authors:** Marit Ilves, Pia Anneli Sofia Kinaret, Joseph Ndika, Piia Karisola, Veer Marwah, Vittorio Fortino, Yuri Fedutik, Manuel Correia, Nicky Ehrlich, Katrin Loeschner, Alexandros Besinis, Joanne Vassallo, Richard D. Handy, Henrik Wolff, Kai Savolainen, Dario Greco, Harri Alenius

**Affiliations:** 1 Human Microbiome Research Program, Faculty of Medicine, University of Helsinki, 00290 Helsinki, Finland; 2 Institute of Biotechnology, University of Helsinki, 00790 Helsinki, Finland; 3 Faculty of Medicine and Life Sciences, University of Tampere, 33100 Tampere, Finland; 4 Biomedicine Institute, University of Eastern Finland, 70211 Kuopio, Finland; 5 PlasmaChem GmbH, 12489 Berlin, Germany; 6 National Food Institute, Technical University of Denmark, 2800 Lyngby, Denmark; 7 Department of Chemical and Biochemical Engineering, Technical University of Denmark, 2800 Lyngby, Denmark; 8 School of Biological and Marine Sciences, Faculty of Science and Engineering, University of Plymouth, Drake Circus, Plymouth, PL4 8AA UK; 9 Plymouth University Peninsula Schools of Medicine and Dentistry, University of Plymouth, John Bull Building, Tamar Science Park, Plymouth, PL6 8BU UK; 10 Finnish Institute of Occupational Health, 00250 Helsinki, Finland; 11 Department of Pathology, University of Helsinki, 00014 Helsinki, Finland; 12 Institute of Environmental Medicine, Karolinska Institutet, 171 77 Stockholm, Sweden

**Keywords:** CuO, Engineered nanomaterial, Health effects, Inflammation, Asthma, Allergic airway inflammation, Risk assessment

## Abstract

**Background:**

Copper oxide (CuO) nanomaterials are used in a wide range of industrial and commercial applications. These materials can be hazardous, especially if they are inhaled. As a result, the pulmonary effects of CuO nanomaterials have been studied in healthy subjects but limited knowledge exists today about their effects on lungs with allergic airway inflammation (AAI). The objective of this study was to investigate how pristine CuO modulates allergic lung inflammation and whether surface modifications can influence its reactivity.

CuO and its carboxylated (CuO COOH), methylaminated (CuO NH_3_) and PEGylated (CuO PEG) derivatives were administered here on four consecutive days via oropharyngeal aspiration in a mouse model of AAI. Standard genome-wide gene expression profiling as well as conventional histopathological and immunological methods were used to investigate the modulatory effects of the nanomaterials on both healthy and compromised immune system.

**Results:**

Our data demonstrates that although CuO materials did not considerably influence hallmarks of allergic airway inflammation, the materials exacerbated the existing lung inflammation by eliciting dramatic pulmonary neutrophilia. Transcriptomic analysis showed that CuO, CuO COOH and CuO NH_3_ commonly enriched neutrophil-related biological processes, especially in healthy mice. In sharp contrast, CuO PEG had a significantly lower potential in triggering changes in lungs of healthy and allergic mice revealing that surface PEGylation suppresses the effects triggered by the pristine material.

**Conclusions:**

CuO as well as its functionalized forms worsen allergic airway inflammation by causing neutrophilia in the lungs, however, our results also show that surface PEGylation can be a promising approach for inhibiting the effects of pristine CuO. Our study provides information for health and safety assessment of modified CuO materials, and it can be useful in the development of nanomedical applications.

**Electronic supplementary material:**

The online version of this article (10.1186/s12989-019-0309-1) contains supplementary material, which is available to authorized users.

## Background

Nanotechnology is a rapidly expanding field of material manipulation. It focuses on improving physicochemical characteristics of different materials and thereby offers numerous possibilities for product development in several industrial sectors. Metal oxides are one of the most abundantly produced types of engineered nanomaterials (ENM) with production volumes of up to thousands of tons every year [1]. CuO nanomaterials are appealing due to their electrical, optical, magnetic and biocidal features, thus they are produced for variety of industrial and commercial applications, such as electronic chips, solar cells, lithium batteries, paints, processed wood and plastics [2–4]. Because of their antimicrobial properties, CuO nanomaterials are used or could be utilized in food packaging, wound dressings, skin products and textiles [2–4]. An important innovation in health care is CuO-containing bed sheets that reduce the occurrence of hospital-acquired infections and thus decrease health care costs in medical facilities [4]. In addition, CuO ENM can be potentially used in nanomedicine as anti-cancer and bioimaging agents [5, 6].

Asthma is a pulmonary disease that affects over 330 million people worldwide and its prevalence is rising rapidly [7]. It is characterized by chronic airway inflammation, reoccurring episodes of airway obstruction, airway hyperresponsiveness (AHR), mucus overproduction and airway remodeling [8, 9]. Asthma has multiple forms and it can be caused by several environmental factors in combination with over 100 susceptibility genes [8]. The extrinsic phenotype (also known as allergic or atopic asthma) starts early on in life and is triggered by an allergen, such as pollen or pet dander. Another pathway of asthma (intrinsic, non-allergic) is independent of Th2 cells and adaptive immunity but relies on the presence of neutrophils and IL-17, which is a neutrophilic chemotactic factor. This “neutrophilic asthma” is associated with nonallergic stimuli, such as cigarette smoke, cold air or pollutants. Pulmonary neutrophilic inflammation is also a major characteristic of patients with severe, steroid non-responding asthma [8–10].

The most common phenotype of asthma found in children and half of adults is atopic in nature [11]. The classical asthmatic response can be divided into early and late phase reactions. During the immediate, early phase response, antigen contact causes local mast cell activation and release of their mediators that ultimately culminates in an acute asthmatic attack. The late-phase response arrives several hours later and can last for prolonged periods of time [12]. Pathomechanistically, the allergens cause a complex immune response that starts with the activation and differentiation of allergen-specific CD4+ T helper (Th) 2 cells. Th2 lymphocytes mediate their functions by releasing Th2 cytokines IL-4, IL-5, and IL-13 [11]. IL-4 switches B-cell antibody production to IgE that subsequently activates the high-affinity IgE receptor FcεRI-carrying mast cells, eosinophils and basophils. IL-5 recruits eosinophils into the lungs and IL-13 triggers AHR and mucus hyperproduction which are classical hallmarks of allergic asthma [9].

As for many nanomaterials, inhalation is one of the most significant routes of CuO exposure. Pulmonary exposure to CuO nanomaterials has been investigated previously in healthy subjects [13, 14], but there is only limited information about the effects of the material on asthma as well as on allergic airway inflammation (AAI) – a condition preceding the chronic disease [2]. Using a mouse model of ovalbumin (OVA)-induced AAI (Additional file 5: Figure S1), the aim of this study was to investigate the adverse effects of nano-sized core CuO (CuO) on the pulmonary condition, and to understand whether the carboxylated (CuO COOH), methylaminated (CuO NH_3_) and PEGylated (CuO PEG) derivates of CuO influence its reactivity.

## Results

### Particle characterisation and the total measured copper content in the test materials

The total measured Cu concentration in the original powders of the different test materials is presented in Table 1, along with the details of purity, primary particle size, surface area and zeta potential of the materials investigated. On a mass basis of each material, the Cu content of each material varied according to the proportion of mass attributed to the coating. Consequently, less total Cu was measured in the coated ENMs relative to the uncoated form (Table 1). For the coated CuO NPs, the NH_4_^+^-coated NPs were found to contain the highest measured fraction of Cu (0.52), followed by the COOH-coated NPs (0.43) and least Cu in the PEG-coated NPs (0.29). Example images of the nanomaterials with particle size distributions of dispersions measured by nanoparticle tracking analysis are shown in Additional file 6: Figure S2 and Additional file 7: Figure S3. The primary particle sizes of the powders, as measured by transmission electron microscopy (TEM), did not exceed the manufacturer’s reported size range (10–20 nm). Following dispersion of the test materials in ultrapure water, the mean hydrodynamic diameters of the aggregates were measured by nanoparticle tracking analysis, and were: 41 nm in the uncoated CuO NPs, 46 nm in the ammonium-coated CuO NPs, 121 nm in the COOH-coated CuO NPs and 100 nm in the PEG-coated CuO NPs (Table 1). Similar sizes of aggregates were formed for the respective particles in Krebs physiological saline (Table 1). The dialysis experiments revealed some Cu dissolution from the different CuO NPs in ultrapure water. The dialysis curves are shown in Additional file 8: Figure S4. The dissolution rate of the uncoated CuO NPs was low (1.68 μg Cu h^− 1^, Table 1), but in comparison, all the coated CuO NPs had higher dissolution rates; greater than 18 μg Cu h^− 1^ in ultrapure water (Table 1). However, the rates were still micromolar, and even the highest rates would only equate to maximally around 6–9% of the total metal being released every hour in water. The dissolution rates for the ENM in Krebs physiological saline were much lower than the equivalent material in ultrapure water (Table 1), and represented less than 3% of metal being released in an hour. Thus, it is expected that the materials will remain in particulate form in physiological saline over 24 h.

**Table 1:** Characterization of the CuO-containing ENMs from the original powders, adapted from Vassallo et al. [15]

Test material (Supplier)	Manufacturer’s information	^c^Measured primary particle size (nm)	^d^Measured hydrodynamic diameter in ultrapure water or Krebs saline (nm)	^e^Total measured copper concentration (mg l^-1^)	^f^Percentage of nominal concentration (%)	^g^Measured copper fraction in coated CuO NPs	^h^Metal dissolution rate in ultrapure water or Krebs saline (μg Cu h^-1^)	^i^Zeta potential in ultrapure water (mV)
^a^CuO NPs uncoated, CAS 1317-38-0 (PlasmaChem GmbH, Lot YF1309121)	99% purity; diameter, 10 - 20 nm; ^b^surface area 42 ± 2 m^2^ g^-1^	12.00 ± 0.37	41 ± 28 (water) 62 ± 50 (Krebs)	287.1 ± 14.4	89.7 ± 4.5	--	1.68 (water) 1.21 (Krebs)	14.0 ± 1.2
^a^CuO NPs COOH-coated, CAS 1317-38-0 (PlasmaChem GmbH, Lot YF140114)	99% purity; diameter, 10 - 20 nm; ^b^surface area, 7.4 ± 0.5 m^2^ g^-1^	6.45 ± 0.16	121 ± 91 (water) 128 ± 58 (Krebs)	154.3 ± 6.9	-	0.43 ± 0.02	69.12 (water) 18.15 (Krebs)	- 7.3 ± 0.5
^a^CuO NPs NH_4_^+^-coated, CAS 1317-38-0 (PlasmaChem GmbH, Lot YF140114)	99% purity; diameter, 10 - 20 nm; ^b^surface area, 6.1 ± 0.5 m^2^ g^-1^	9.53 ± 0.22	46 ± 36 (water) 96 ± 75 (Krebs)	185.9 ± 7.2	-	0.52 ± 0.02	18.6 (water) 12.22 (Krebs)	27.7 ± 0.5
^a^CuO NPs PEG-coated, CAS 1317-38-0 (PlasmaChem GmbH, Lot YF140114)	99% purity; diameter, 10 - 20 nm	7.46 ± 0.42	100 ± 36 (water) 189 ± 113(Krebs)	105.0 ± 3.5	-	0.29 ± 0.01	52.02 (water) 17.44 (Krebs)	- 16.8 ± 0.4

^a^Supplied as dry powders, bespoke design and production of spherical particles for the NANOSOLUTIONS project *via* Alexei Antipov, PlasmaChem GmbH

^b^Brunauer–Emmett–Teller (BET) surface area values (mean ± one standard deviation, *n* = 3) from NANOSOLUTIONS project conducted by A. Besinis

^c^Based on transmission electron microscopy (TEM) images of CuO ENMs from a 100 mg l^-1^ Cu stocks in Milli-Q water where data are mean ± standard error of the mean (S.E.M) with *n* = 60 measurements

^d^Particle size distribution measurements (mean ± one standard deviation, *n* = 3) by Nanoparticle tracking analysis (NTA) on 100 mg l^-1^ Cu ENM stocks in Milli-Q water or Krebs physiological saline at pH 7.4

^e^Data are means ± S.E.M (*n* = 3 replicates) of total measured copper concentration by ICP-OES following *aqua regia* acid digestion of the dry powders, and after normalisation to an initial 0.02 g weight of material; Cupric oxide nanoparticles (CuO NPs)

^f^With a 0.8 fraction of copper by weight in uncoated CuO NPs

^g^Relative to the measured copper content in the uncoated CuO NPs

^h^Maximum slope from rectangular hyperbola function of curve fitting used to estimate the maximum rate of dissolution of copper from the dialysis experiments, in triplicate

^i^Zeta potential of CuO dispersions in ultrapure water (pH 5) as an average of five separate measurements.- Not possible to calculate from the manufacturer’s information on material composition; -- Data not applicable to the test material. The Krebs physiological saline data are from Besinis and Handy, unpublished

### Exposure to CuO nanomaterials induced significant neutrophil migration into the lungs of phosphate-buffered saline (PBS)- and OVA-challenged mice

**Fig. 1:**
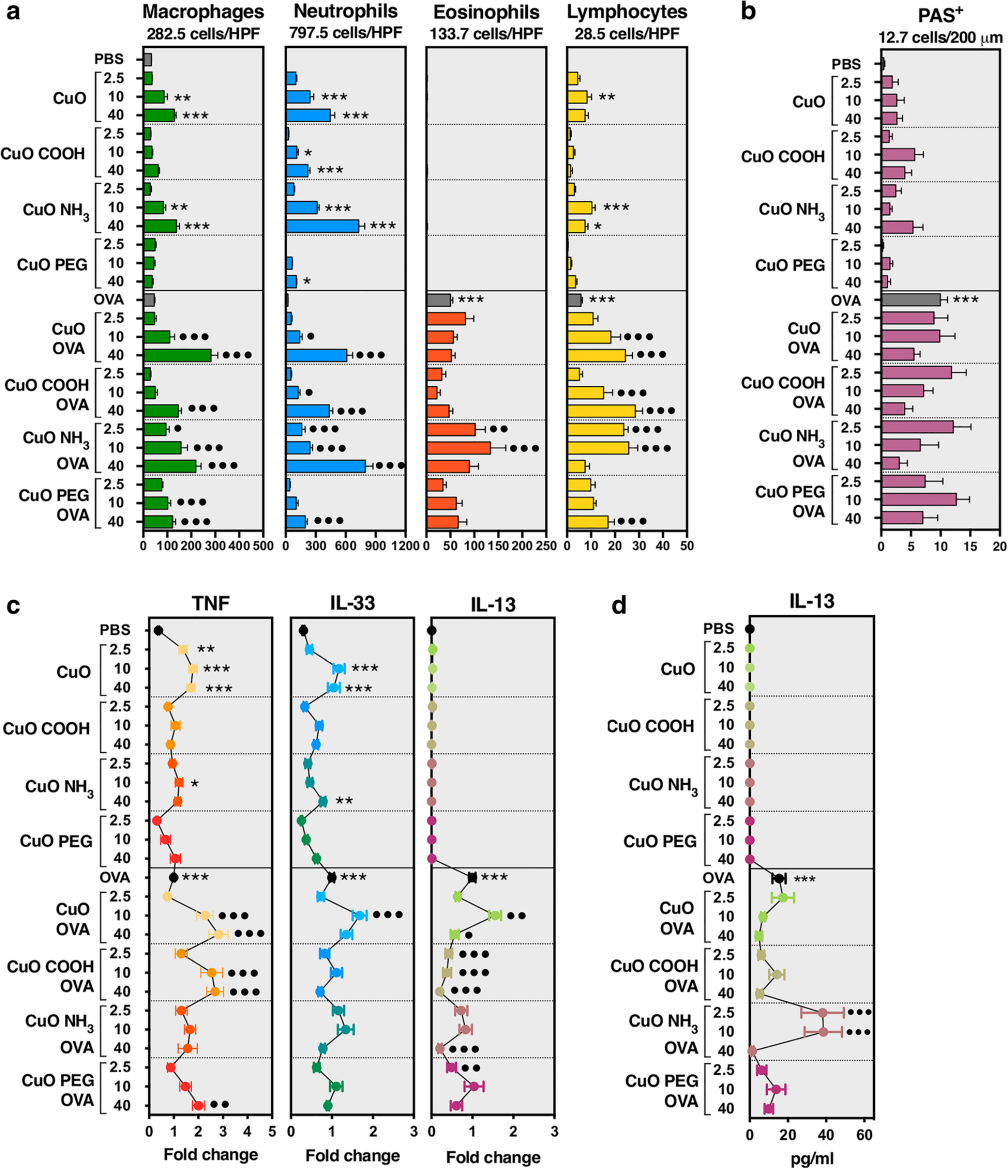
Cell counts, expression and release of (pro-)inflammatory markers following exposure to CuO materials**.** BALB/c mice were sensitized ip to OVA/Alum on day 1 and 10, and exposed repeatedly to 2.5, 10 or 40 μg/mouse of CuO nanomaterials dispersed in PBS with or without OVA by oropharyngeal aspiration after a 10-day recovery period. **a** BAL cell counts showed the ability of CuO materials to trigger an increase in the number of macrophages, lymphocytes and especially neutrophils into the airways of both PBS- and OVA-challenged mice whereas eosinophils were detected only in OVA-challenged groups. **b** PAS staining revealed that CuO materials did not activate mucin-production in goblet cells. **c** Pro-inflammatory cytokines TNF and IL-33 were expressed in PBS-challenged as well as OVA-challenged mice while Th2 type cytokine IL-13 was expressed only in OVA-challenged mice. **d** IL-13 protein was detected in BAL supernatants by ELISA also only in OVA-challenged mice. Results in **a-b** are shown with the highest number of cells marked on top of each plot. Columns and error bars represent mean values ± standard error of mean (SEM). Statistically significant differences between experimental groups and PBS-challenged control mice are marked with “*” whereas the ones between experimental groups and OVA-challenged control mice are marked with “•”. */•*P* < 0.05; **/••*P* < 0.01; ***/•••*P* < 0.001. HPF, high power field; PAS, periodic acid–Schiff

**Fig. 2:**
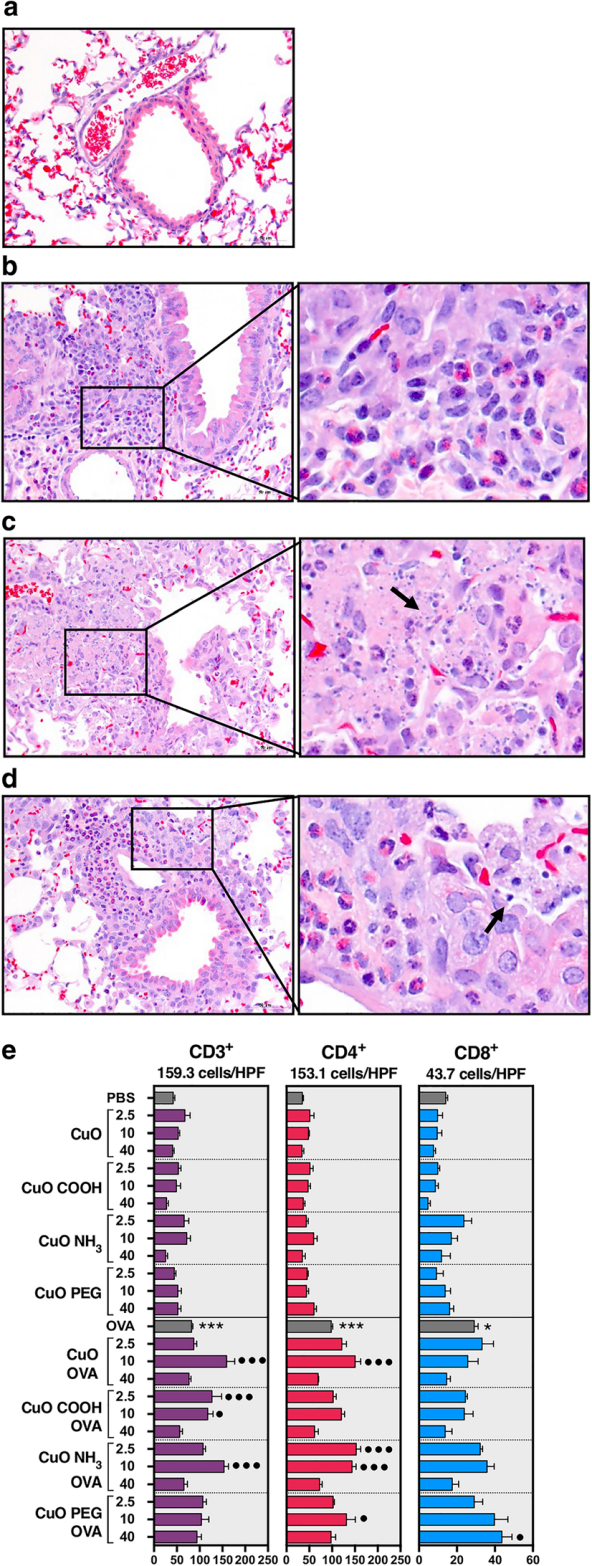
Histological and immunohistochemical evaluation of the lung tissue after exposure to CuO nanomaterials**.** BALB/c mice were sensitized ip to OVA/Alum on day 1 and 10, and exposed repeatedly to 2.5, 10 or 40 μg/mouse of CuO nanomaterials dispersed in PBS with or without OVA by oropharyngeal aspiration after a 10-day recovery period. H&E-stained lung tissue of **a** a PBS-challenged control, **b** OVA-challenged, **c** PBS-challenged and 40 μg of core CuO-exposed, and **d** OVA-challenged and 40 μg of core CuO-exposed mouse. The presence of eosinophils in OVA-challenged mice and neutrophils accompanied with nuclear dust in CuO-treated mice were found. E, The number of CD3+, CD4+ and CD8+ T cells in the lung tissue. Images **a-d** are shown at × 400 magnification with a 50-μm scale bar. Arrows in the insets indicate the location of nuclear dust. Counts of T cell subtypes in **e** are shown as positive cells per high power field (HPF) with the highest number of cells marked on top of each plot. Columns and error bars represent mean values ± standard error of mean (SEM). Statistically significant differences between experimental groups and PBS-challenged control mice are marked with “*” whereas the ones between experimental groups and OVA-challenged control mice are marked with “•”. */•*P* < 0.05; ***/•••*P* < 0.001

To characterize the pulmonary effects of CuO materials, inflammatory cell influx in bronchoalveolar lavage (BAL) and pathological changes in lung tissue were evaluated along with mRNA expression analysis of cytokines in tissue samples. Development of AAI in our model was evidenced by increased number of eosinophils and lymphocytes in BAL, activation of goblet cells in lung tissue and up-regulation of pro-inflammatory TNF, pro-allergic IL-33 and Th2 type IL-13 in the lungs of OVA-challenged compared with PBS-challenged mice (OVA versus PBS group in Fig. 1). Furthermore, OVA-challenged mice exhibited eosinophils as well as elevated number of CD4+ T cells in the lung tissue (OVA versus PBS in Fig. 2a-b, e).

CuO materials tested in our study induced a significant neutrophil influx into the airways of PBS- as well as OVA-challenged mice (Fig. 1a). The materials did not influence the number of eosinophils in BAL nor mucus-producing goblet cells in the lung tissue of OVA-challenged mice, except in case of CuO NH_3_ whose exposure triggered additional migration of eosinophils into the airways (Fig. 1a-b). Co-exposure to OVA and CuO materials had an additive (mostly dose-dependent) effect on the number of macrophages and lymphocytes (Fig. 1a). Total number of BAL cells and relative proportion of differential immune cells is presented in Additional file 1: Table S1.

CuO nanomaterials increased the expression of pro-inflammatory TNF and pro-allergic IL-33 but did not up-regulate IL-13 expression in PBS-challenged mice, as compared to specific controls (Fig. 1c). TNF and IL-33 levels were increased especially in response to CuO in both PBS-challenged as well as OVA-challenged in mice. In OVA-challenged mice, CuO nanomaterials rather suppressed the expression of IL-13 compared with OVA controls (Fig. 1c). IL-13 was measured also at the protein level in BAL supernatants and no significant changes were seen in OVA-challenged groups, except in a case of CuO NH_3_ that had increased IL-13 protein release at doses 2.5 and 10 μg/mouse (Fig. 1d).

Histological assessment of mice treated with CuO nanomaterials, revealed the presence of inflammatory areas and nuclear dust (Fig. 2a-d; Additional file 9: Figure S5). These changes were seen in both PBS- and OVA-challenged mice treated with 10 and 40 μg/mouse of CuO, CuO COOH and CuO NH_3_. Inflammation was milder at 10 μg/mouse in CuO PEG-exposed mice (Additional file 9: Figure S5A) whereas the effects were comparable to the ones of other CuO materials at 40 μg/mouse. Nuclear dust was especially evident in the lung tissue at the highest dose of 40 μg/mouse, at which it was observed in response to all ENM (Fig. 2c-d). Since all materials exhibited similar changes at the highest dose, quantification of the inflammatory areas and nuclear dust was performed at dose 10 μg/mouse. The core material and CuO PEG only were selected for evaluation because the other surface-functionalized materials had equivalent effects on the lung tissue at this dose as CuO. It was found that exposure to CuO triggered or enhanced inflammation and caused the formation of nuclear dust in both OVA- and PBS-challenged mice as compared with their respective controls (Additional file 9: Figure S5B). CuO PEG-induced inflammation in PBS-challenged mice was minor compared with PBS controls and significantly milder compared to the inflammation caused by CuO. Similar result appeared also in OVA-challenged mice but the difference between the groups of CuO and CuO PEG was statistically insignificant. Nuclear dust was observed only in CuO- but not in CuO PEG-treated mice (Additional file 9: Figure S5C).

Immunohistochemical staining of lung tissue revealed no increased influx of T cell subtypes in PBS-challenged mice. However, increase of CD3+ T cells in OVA-challenged mice was seen after treatment with all CuO materials, especially at the dose of 10 μg/mouse, except with CuO PEG (Fig. 2e). Further characterization showed that this increase was derived from the elevated presence of CD4+ T cells since the numbers of CD8+ T cells remained generally at similar levels as in OVA-challenged mice.

### Transcriptome analysis revealed that CuO PEG has a lower potential in triggering changes at gene expression level than other tested materials

**Fig. 3:**
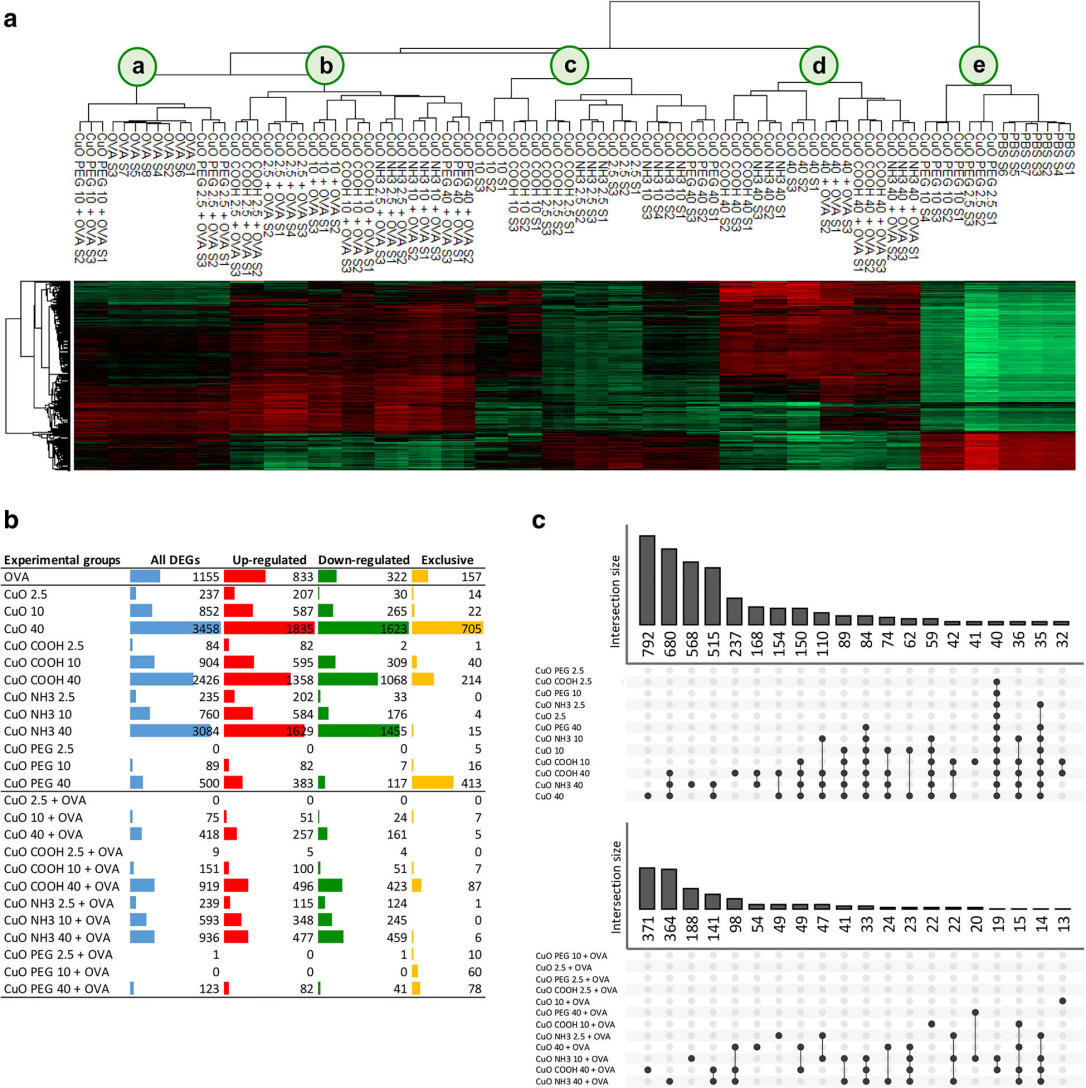
Differential gene expression analysis**. a** Heat map of top 500 differentially expressed genes (linear FC > |1.5|, adjusted *P* value < 0.05) in each analyzed lung tissue sample of PBS- and OVA-challenged mice exposed to CuO nanomaterials by oropharyngeal aspiration (2.5, 10 and 40 μg/mouse). Z-score normalized log2 intensity values were used as input for hierarchical clustering. Red color indicates a higher expression while green refers to a lower expression. **b** Numbers of total, up-regulated, down-regulated and exclusively differentially expressed genes (DEGs; linear FC > |1.5|, adjusted *P* value < 0.05) in lung tissue after exposure to CuO nanomaterials of PBS- and OVA-challenged mice versus the corresponding control mice. **c** UpSet plots showing 20 largest intersections of DEGs that are either specific to a treatment or shared between experimental groups among PBS and OVA-challenged mice. The upper bar chart indicates the number of DEGs in each intersection

Hierarchical clustering of top 500 differentially expressed genes (DEGs) showed that lung tissue of PBS-challenged mice exposed to the two lower doses (2.5 and 10 μg/mouse) of CuO PEG shared similar expression patterns with their controls (cluster e in Fig. 3a). The same trend was also seen among OVA-challenged mice (cluster a in Fig. 3a). Samples from mice treated with lower doses of all other materials and the highest dose of CuO PEG clustered together within both, PBS-challenged and OVA-challenged mice (clusters b-c in Fig. 3a). Regardless of the allergen challenge, mice exposed to higher doses of all materials except CuO PEG shared a similar expression pattern (cluster d in Fig. 3a).

Number of DEGs in each experimental group compared with their respective controls revealed that more changes took place in PBS-challenged than in OVA-challenged mice (Fig. 3b, Additional file 2: Table S2). Every material triggered a dose-dependent response as the number of DEGs was increasing across the doses. CuO PEG induced notably less changes compared with other CuO materials which supports the finding seen on the heatmap. Venn comparisons of DEG sets of experimental groups revealed that the highest number of DEGs were exclusive or shared between higher doses of CuO, CuO COOH and CuO NH_3_ in both PBS-challenged and OVA-challenged mice (Fig. 3c).

To validate the microarray data, monocyte chemoattractants CCL2 (MCP-1) and CCL7 (MCP-3), and eosinophil-recruiting CCL11 (eotaxin-1) were chosen for reanalysis by real-time quantitative polymerase chain reaction (PCR). The PCR-based expression levels were highly correlating (Pearson’s r ≥ 0.95) with the transcriptomics data (Additional file 10: Figure S6). Furthermore, CCL2 was measured at protein level in BAL supernatants and its levels were highly similar to those obtained by microarray and PCR. In addition, several biomarkers linked to AAI were also identified in the microarray-based transcriptomics data to present the differences between PBS- and OVA-challenged control mice (Additional file 11: Figure S7).

### Irrespective of differentiating transcriptome profiles, CuO, CuO COOH and CuO NH_3_ displayed commonalities at pathway level

**Fig. 4:**
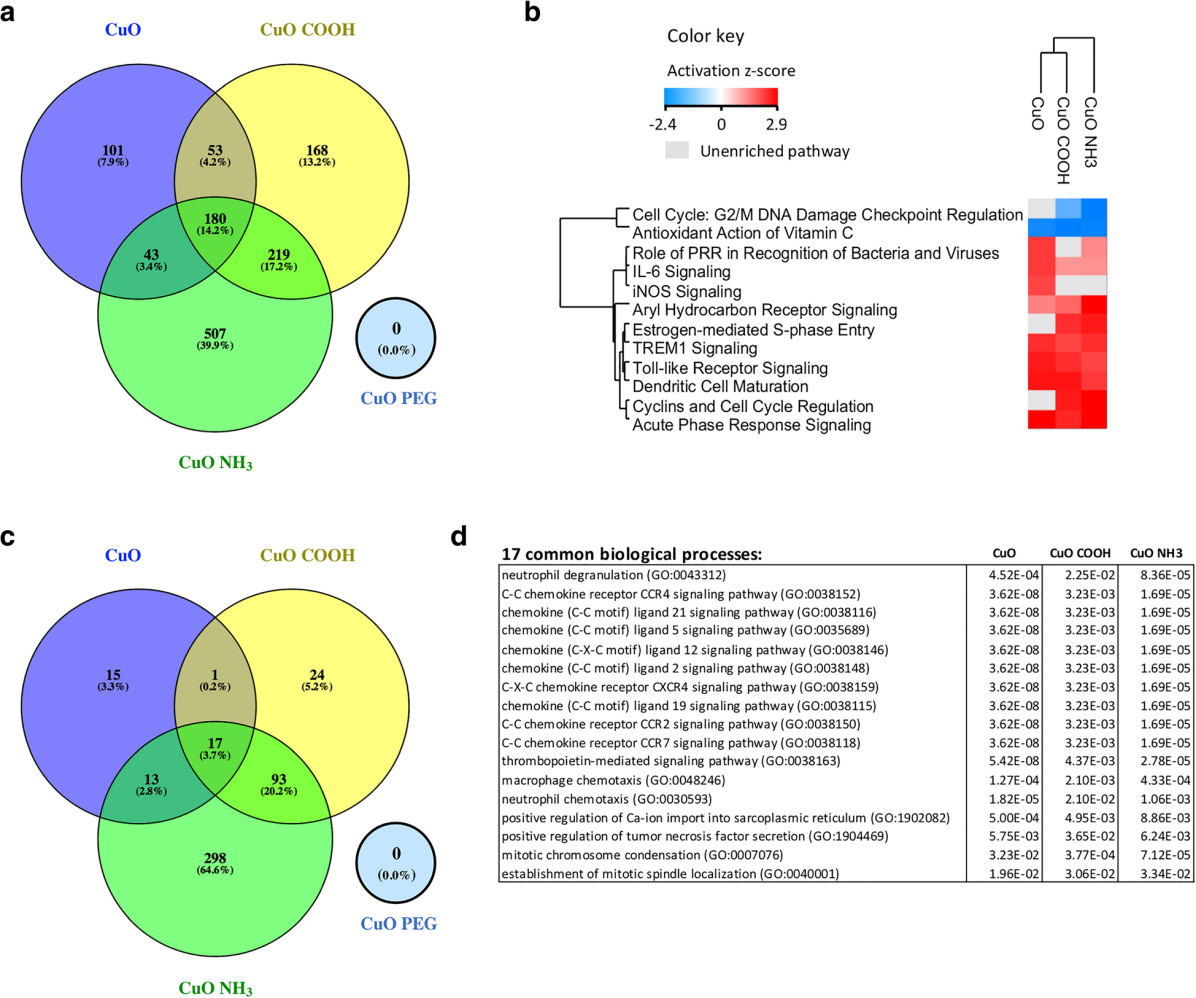
General profiling of canonical pathways and biological processes in PBS-challenged mice after CuO, CuO COOH and CuO NH_3_ exposure**.** Experimental groups of exposed PBS-challenged mice were merged based on the test material, and their transcriptomic profiles were compared against the one of PBS-challenged controls. **a** A Venn distribution of the number of DEGs (linear FC > |1.5|, adjusted *P* value < 0.05) in lungs of PBS-challenged mice exposed to CuO nanomaterials by oropharyngeal aspiration (2.5, 10 and 40 μg/mouse). **b** A heat map showing the activation z-scores of IPA canonical pathways filtered with z-score > 2 and -log(*P* value) > 2 across the materials. **c** Venn comparison of significantly enriched biological processes (adjusted *P* value < 0.05) obtained from analyses of DEG sets shown in (**a**). **d** 17 biological processes were commonly enriched by DEGs from the CuO, CuO COOH and CuO NH_3_ exposures

Excluding dose as a variable, we combined experimental groups of PBS-challenged or OVA-challenged mice that were treated with the same CuO material and compared their transcriptomic profiles against the ones of their corresponding controls to explore general differences between the materials at gene expression level (Fig. 4, Additional file 12: Figure S8, Additional file 3: Table S3). CuO PEG did not produce any DEGs in mice challenged with PBS or OVA, while the other three materials elicited specific transcriptomic changes in both phenotypes. In PBS-challenged mice, 7.9, 13.2 and 39.9% of the DEGs were identified as unique to CuO, CuO COOH and CuO NH_3_ respectively, while 14.2% of the DEGs were shared between all three materials (Fig. 4a). A similar trend – where the most unique transcriptomic responses originated from CuO NH_3_ – was also observed in transcriptome of OVA-challenged mice (Additional file 12: Figure S8), but no functionally (in)activated biological processes could be identified based on Ingenuity™ pathway analysis (IPA) [16]. On the other hand, in PBS-challenged mice, the material-type-specific DEGs were predicted to activate/inactivate several pathways where the extent of activation/deactivation varied according to the material surface chemistry (Fig. 4b). All three materials suppressed Cell Cycle at G2/M DNA Damage Checkpoint Regulation and Antioxidant Action of Vitamin C pathway, for which the lowest degree of inhibition was predicted for core CuO DEGs. Similar surface-chemistry-based bioreactivity was also observed for the predicted activation of several innate immunity and pro-inflammatory pathways (Fig. 4b; IPA comparison analysis). Among others, Aryl Hydrocarbon Receptor Signaling (least activated by core CuO) and Dendritic Cell Maturation pathways (least activated by CuO NH_3_) were activated.

To study similarities of the biological processes, EnrichR tool was used [17, 18]. In PBS-challenged mice, 17 processes were commonly enriched in response to CuO, CuO COOH and CuO NH_3_ (Fig. 4c). Majority of these were related to cytokine/chemokine signaling, but other inflammatory processes, such as macrophage and neutrophil chemotaxis, and cell division overlapped between the materials as well (Fig. 4d). In OVA-challenged mice, only few DEGs were obtained (Additional file 12: Figure S8A), however, enrichment analysis revealed one common biological process, i.e. neutrophil degranulation (Additional file 12: Figure S8B) that was seen also in PBS-challenged mice.

### PEGylation modulates bioreactivity of pristine CuO via predicted suppression of inflammatory pathways

**Fig. 5:**
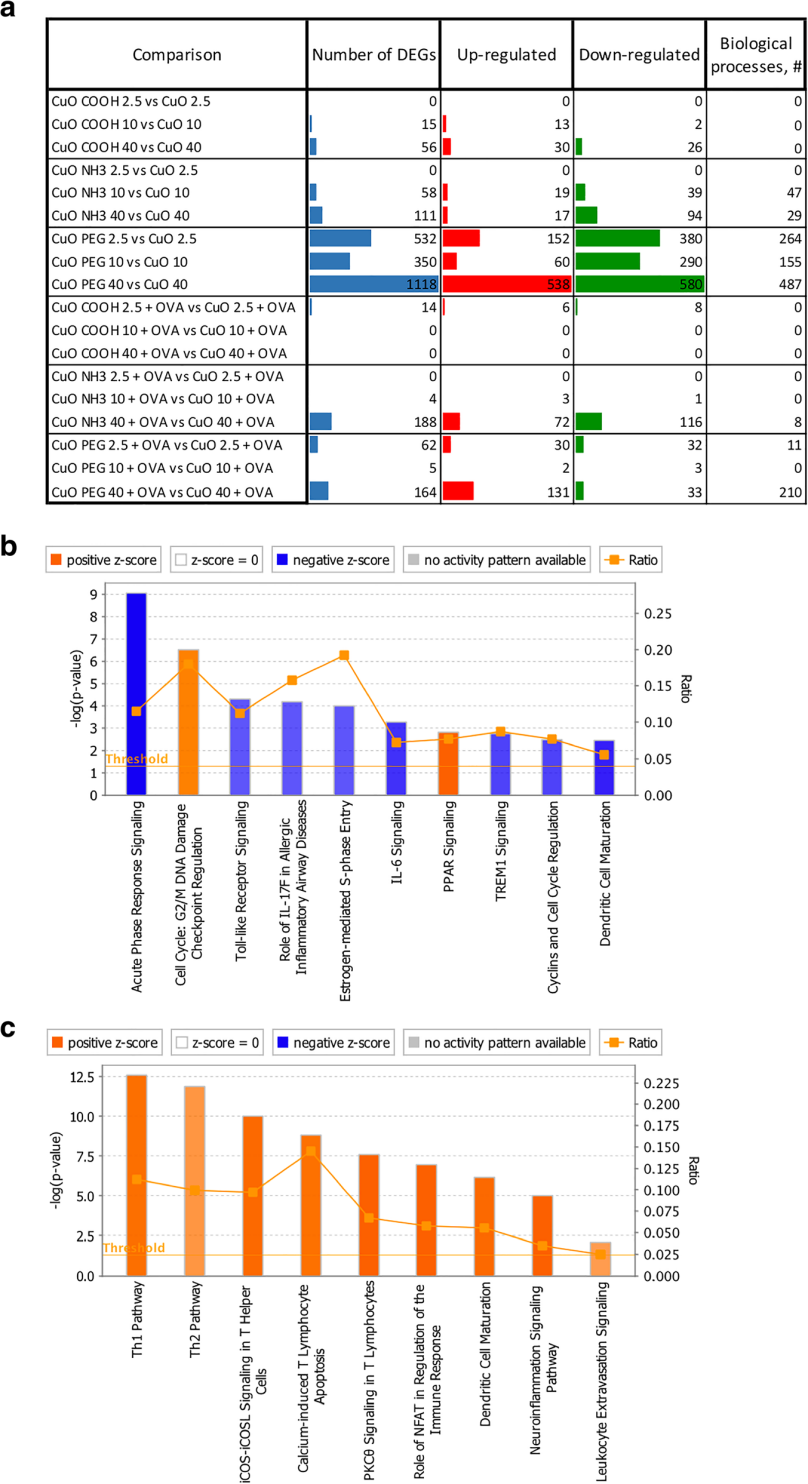
Modulatory effects of surface functional groups on CuO bioreactivity**.** As shown in (**a**), at each dose, the experimental group of every functionalized material was compared against core CuO in PBS- or OVA-challenged mice. The most drastic effect was induced by PEGylation, as seen from the Number of DEGs and enriched biological processes (**a**). The top canonical pathways enriched by down-regulated genes in PBS mice exposed to 2.5 μg of CuO PEG as compared with core CuO at the same dose, or enriched by up-regulated genes in OVA-challenged mice exposed to 40 μg of CuO PEG as compared with core CuO at the same dose are shown in (**b**) and (**c**) respectively. **b-c** The x-axis represents the pathways identified. The y-axis on left shows the -log(*P* value) calculated based on Fisher’s exact test. Threshold level is set on a *P* value of 0.05. The y-axis on right represents a ratio between a number of DEGs in a given pathway with a cut-off -log(*P* value) > 2, divided by total number of genes belonging to the reference gene set of the pathway. The orange- and blue-colored bars indicate predicted pathway activation or inhibition, respectively

To investigate how surface functionalization affects the effects of core CuO, each dose of every modified material was compared against the corresponding dose of core CuO (Fig. 5, Additional file 4: Table S4). Numbers of DEGs showed that CuO COOH and CuO NH_3_ triggered notably less changes than CuO PEG at all doses. Enrichment analysis by PANTHER [19] revealed that DEGs of CuO COOH and CuO NH_3_ groups resulted in none or few significantly enriched biological processes (Fig. 5a). In contrast, a large number of biological processes were enriched by the set of DEGs in treatment groups of CuO PEG. Therefore, CuO PEG was chosen for further analysis to understand how the surface modification alters the effect of the core material.

Up- and down-regulated genes derived from CuO PEG groups were analyzed separately. It was found that in PBS-challenged mice at the lowest dose of 2.5 μg/mouse, only down-regulated genes resulted in significantly enriched biological processes. Majority of these processes categorized under inflammatory response, cell and nuclear division, positive regulations of immune system process and regulation of cytokine production (Additional file 13: Figure S9). Causal analysis using IPA’s Ingenuity Knowledge Base further confirmed that the down-regulated genes predictively suppress several innate immunity and pro-inflammatory pathways (Fig. 5b; dose 2.5 μg/mouse).

In contrast, immune system related processes such as leucocyte activation, cytokine production, positive regulation of cell-cell adhesion and immune system process were significantly enriched by up-regulated genes of CuO PEG at the highest dose (40 μg/mouse) (Additional file 14: Figure S10A). Down-regulated genes enriched processes belonging to negative regulation of peptidase activity, mitotic cell cycle, nuclear division, response to wounding and positive regulation of cell proliferation (Additional file 14: Figure S10B). Enriched inflammatory processes represented by the up-regulated genes in OVA-challenged mice exposed to the highest dose of CuO PEG were inflammatory response, leucocyte activation, immune system process and positive regulation of cytokine production (Additional file 15: Figure S11). Furthermore, IPA causal analysis indicated that the direction of gene expression change was consistent with activation of these canonical pathways (Fig. 5c; dose 40 μg/mouse).

### Activation of pathways affected by CuO materials was more distinguishable in PBS-challenged than in OVA-challenges mice

To understand how CuO materials modulate OVA-induced AAI, DEG sets of PBS-challenged mice were compared to the ones of OVA-challenged mice. Overlaps of DEGs were minor at the lowest concentration for all materials, however, majority of the DEGs in OVA-challenged groups were present also in PBS-challenged mice (Additional file 16: Figure S12A). Hierarchical clustering of canonical pathways revealed two main clusters: groups of PBS- and OVA-challenged mice exposed to lower doses (2.5 and 10 μg/mouse) of all CuO materials and to the highest dose (40 μg/mouse) of CuO PEG, and groups of PBS- and OVA-challenged mice treated with the highest dose of all materials except CuO PEG (Additional file 16: Figure S12B). Majority of the pathways in OVA-challenged mice that were exposed to lower doses of CuO materials, were unenriched or did not obtain an activation score due to low number of DEGs. A subcluster of canonical pathways related to T cell regulation (Th1 and Th2 Pathway, CD28 Signaling in T Helper Cells, PKC Signaling in T Lymphocytes, iCOS-iCOSL Signaling in T Helper Cells and Calcium-induced T Lymphocyte Apoptosis) were non-activated or slightly up-regulated at lower doses of materials in PBS-challenged mice while at the higher dose, these pathways were commonly suppressed regardless of the disease status (Additional file 16: Figure S12B).

With the exception of OVA-challenged mice treated with lower doses of the ENM, administration of CuO materials suppressed cell cycle in G2/M DNA damage checkpoint. Other cell cycle pathways, such as Estrogen-mediated S-phase Entry, Cyclins and Cell Cycle Regulation and Mitotic Roles of Polo-Like Kinase were activated by exposure to CuO materials (Additional file 16: Figure S12B). CuO materials activated also inflammatory pathways (e.g. Role of PRR in Recognition of Bacteria and Viruses, Dendritic Cell Maturation, IL-6 Signaling, Role of IL-17F in Allergic Inflammatory Airway Diseases, Aryl Hydrocarbon Receptor Signaling, and Acute Phase Response Signaling), all of whose activation appeared generally more distinguishable in PBS-challenged mice than in OVA-challenged mice.

### Pathways influenced by CuO materials do not dramatically differ in PBS- and OVA-challenged mice

To explore whether CuO materials modulate immune responses differently depending on the pulmonary condition, activation scores of the canonical pathways enriched in PBS- and OVA-challenged groups were compared. Since the DEG sets of OVA-challenged mice exposed to lower doses of CuO materials were small in number (Additional file 16: Figure S12A) and consequently did not result in pathway enrichment or were too few for predicting their activation state (Additional file 16: Figure S12B), canonical pathways enriched in PBS- and OVA-challenged mice at the highest dose (40 μg/mouse) were compared.

Majority of the pathways had a similar activation direction (Additional file 17: Figure S13). Acute phase response signaling and intrinsic prothrombin activation pathway were common among 5 most activated pathways after CuO, CuO COOH and CuO NH_3_ exposure in both PBS- and OVA-challenged mice, whereas Calcium-induced T Lymphocyte Apoptosis, iCOS-iCOSL Signaling in T Helper Cells, Phospholipase C Signaling and PKC Signaling in T Lymphocytes were common among 5 most inhibited pathways. Anti-correlated pathways in PBS- versus OVA-challenged mice occurred after CuO COOH (Dendritic Cell Maturation, Eicosanoid Signaling and Antioxidant Action of Vitamin C) and CuO NH_3_ treatment (TREM1 Signaling), however, their activation state had a z-score ≤ |2| and thus, cannot be considered necessarily (in)activated (Additional file 17: Figure S13B-C). DEG set of OVA-challenged mice after CuO PEG exposure did not result in an activation or suppression of any pathways, however, several pathways that were also enriched in response to other materials, were activated in PBS-challenged mice treated with CuO PEG (Additional file 17: Figure S13D).

## Discussion

CuO nanomaterials can be incorporated into several broadly used consumer products and it is estimated that worldwide production of CuO nanomaterials will raise from 570 tons in 2014 to 1600 tons by the year 2025 [20]. Hazards of CuO nanomaterials in respiratory tract have been evaluated in different in vitro systems [21–25] as well as in healthy subjects in vivo [13, 14]. However, there is a little knowledge about how these materials might affect individuals with a pre-existing lung disease [2, 10]. Furthermore, there is lack of information whether modifying the surface of core CuO could influence their immunomodulatory potential *in vivo.* We used a mouse model of AAI in which the mice were at first sensitized intraperitoneally (ip) to a mixture of an allergen, OVA, and an adjuvant, aluminium/magnesium hydroxide (Alum), and after an 11-day recovery period, challenged repeatedly with OVA. During the challenge phase, CuO materials were administered to the mice on 4 consecutive days via oropharyngeal aspiration. Herein, we made use of standard genome-wide gene expression profiling, coupled with conventional histopathological and immunological methods, to investigate the mechanisms underlying the modulatory effects of CuO nanoparticles and their carboxylated, methylaminated and PEGylated derivatives on healthy and compromised immune system. The lack of parallel proteome-wide assessment is a general limitation of transcriptome-based mechanistic studies, as mRNA and protein levels may not necessarily correlate [26]. Nonetheless, we complemented mRNA profiling with gene ontology-based enriched pathway analysis, which, though not definitive, still provides insight into the biological processes affected by the observed changes in gene expression. In addition to the transcriptomic changes, we studied histological, cellular and humoral responses on the exposed animals to support and reveal additional information on nanomaterial exposure in the lungs.

Classical asthma is eosinophilic with increased number of CD4+ T cells that produce Th2 type cytokines [27]. Furthermore, patients with asthma experience excessive mucus secretion [28], and their sputum and BAL contain elevated amounts of pro-inflammatory cytokines [29]. Previously conducted studies show that ENM modulate AAI differently. Carbon nanotubes [30–33] and silica [34–36] have been found to aggravate signs of AAI by increasing eosinophil influx, triggering mucus hyperplasia and release of pro-allergic cytokines and chemokines whereas exposure to TiO_2_ [37, 38] during the allergen challenge has been reported to diminish local allergic symptoms. In our study, the tested CuO materials triggered a significant neutrophil influx into the airways of PBS- as well as OVA-challenged mice. Furthermore, we detected features similar to nuclear dust (karyorrhexis) in the lung tissue whose presence has been related to and breakdown of leukocytes, especially neutrophils [39]. Since neutrophils were the predominant cell population in the lungs, the nuclear fragments that we evidenced were likely neutrophil-derived. However, eosinophil migration into the airways and activation of mucus-producing goblet cells in the lung tissue were not affected by the nanomaterials in OVA-challenged mice, except in case of CuO NH_3_ that enhanced the eosinophil influx. In addition, when excluding the response of CuO, pro-allergic IL-33 remained at baseline level. Furthermore, we found an elevated presence of CD4+ T cells in OVA-challenged mice after the nanomaterial exposure. CD4+ lymphocytes producing Th2 cytokines are common for extrinsic asthma phenotype, however, as mRNA of Th2 type IL-13 was mainly suppressed by the materials in our study and not significantly changing at protein level except in response to CuO NH_3_, involvement of other types of CD4+ Th lymphocytes in responses driven by CuO nanomaterials might be possible. For example, it has been demonstrated that exposure to pollutant particles results in Th17-biased immunophenotype accompanied by airway neutrophilia [10] which are, in fact, features of severe asthma [40, 41]. Taken together, our data indicate that CuO nanomaterials do not dramatically affect the hallmark features of allergic airway inflammation, but the materials worsen it by adding another inflammatory feature – pulmonary neutrophilia – to the already existing condition.

To elucidate mechanisms behind the CuO-induced effects, we examined material-triggered changes in healthy lungs and in lungs with AAI in more detail by transcriptomics profiling (NCBI GEO accession number GSE122197). Hierarchical clustering of the most differentially expressed genes revealed that changes in PBS-challenged mice and OVA-challenged mice exposed to the two lower doses (2.5 and 10 μg/mouse) of CuO PEG shared similar expression patterns with their corresponding controls (Fig. 3a). Numbers of DEGs in experimental groups showed that more changes took place in PBS-challenged than in OVA-challenged mice and the number of DEGs increased across the doses of each material reflecting to a dose-dependent response. CuO PEG induced clearly less changes compared with other materials supporting the hierarchical clustering of the individual samples. These data show that CuO PEG has significantly lower potential in triggering changes in both healthy and allergic lungs compared with CuO and its two other functionalized derivates.

In order to explore general differences in the bioreactivity of the materials at the molecular level, we combined experimental groups of PBS-challenged or OVA-challenged mice that were treated with the same CuO material and compared their transcriptomic profiles against those of their corresponding controls. CuO PEG did not produce any DEGs in mice challenged with PBS or OVA, however, comparison of the DEGs of the other materials by IPA revealed that CuO materials in PBS-challenged mice predictively suppressed Cell Cycle at G2/M DNA Damage Checkpoint Regulation and Antioxidant Action of Vitamin C pathways, and concurrently activated several innate immunity and pro-inflammatory pathways. Interestingly, Aryl Hydrocarbon Receptor Signaling and Dendritic Cell Maturation pathways, that were also found predictively activated, have been linked to Th17 and IL-17A dependent immune responses and neutrophilic inflammation in response to particulate matter [10]. We then focused on biological processes involved in the material-triggered responses and saw that 17 processes were commonly enriched by DEG sets of CuO, CuO COOH and CuO NH_3_ in PBS-challenged mice. These were mainly inflammatory processes, several of which were related to neutrophil mobilization and activation. For example, CXCL12 interacts with CXCR4 and affects the release of neutrophils from the bone marrow [42], and CCL2 binds to CCR2 and plays a role in neutrophil adherence, transmigration and activation [43]. Rest of the processes included macrophage chemotaxis, regulation of TNF secretion, cell division and chemokine signaling pathways which were in line with IPA results. Enrichment analysis of DEGs in OVA-challenged mice revealed only neutrophil degranulation (seen also in PBS-challenged mice) as a common biological process between CuO, CuO COOH and CuO NH_3_. Neutrophil degranulation followed by the release of neutrophil extracellular traps is a defense mechanism of innate immune system that can be induced by increased intracellular Ca^2+^ [44, 45]. Furthermore, it is a feature of several inflammatory diseases, like acute lung injury and asphyxia episodes of asthma [44]. The uncovered neutrophil-related pathways and biological processes support histological and cytological findings as well as CCL2 protein levels measured in BAL supernatants, which all revealed that neutrophils play a role in CuO-induced reactions regardless of the allergen challenge.

Having established that the material’s bioreactivity is linked to its surface chemistry, we next sought to investigate how surface functionalization modulates the effects of core CuO. For this, each dose of every modified material was compared against the corresponding dose of core CuO. We found that CuO COOH and CuO NH_3_ triggered notably less changes than CuO PEG based on the number of DEGs. Furthermore, enrichment analysis revealed a large number of biological processes specifically in treatment groups of CuO PEG. We then explored how surface PEGylation alters the effect of the core material. By analyzing up- and down-regulated genes separately, we found that at the lowest dose (2.5 μg/mouse) of CuO PEG in PBS-challenged mice, only down-regulated genes resulted in significantly enriched biological, especially inflammatory, processes and IPA showed that the down-regulated genes predictively suppressed innate immunity and pro-inflammatory pathways. On the contrary, immune system related processes were at the highest dose (40 μg/mouse) significantly enriched by up-regulated genes of CuO PEG and similar trend was observed in OVA-challenged mice. Overall, these data show that surface PEGylation changes the effects induced by pristine material the most. At the low concentration, core CuO is activating inflammatory processes in healthy airways that are suppressed when the material is PEGylated, whereas at the high concentration, core CuO suppresses the inflammatory reactions in healthy as well as in asthmatic lungs while PEGylation of the material inhibits it. These findings suggest that surface PEGylation prevents the effects triggered by core CuO from taking place.

Since equivalent molar doses of Cu could not be calculated in this study due to uncertainty of the stoichiometry and the technical challenges in measuring the mass attributed to the coating [46], equivalent mass of the whole material was used for dosing which resulted in differing amounts of Cu in the administered doses of the nanomaterials. Although it may have had an effect on the outcome, it is very unlikely that the Cu fraction in the materials (Table 1) was entirely responsible for the ranking of the inflammatory response. The uncoated CuO contained twice as much Cu metal than CuO COOH and CuO NH_3_, but it was not twice as hazardous. Similarly, CuO NH_3_ or CuO COOH had twice as much Cu metal than CuO PEG but when comparing the number of neutrophils – the most dramatically changed endpoint of this study, these ENM triggered more than two times higher influx of the cells than CuO PEG. In any event, ranking the inflammation by Cu content of the materials would assume metal ion toxicity.

Earlier studies have underlined the importance of extracellular and intracellular dissolution of ENM in biological systems [47]. While significant amount of extracellularly dissolved metal ions can contribute to material toxicity, it has been observed that certain nano-sized metal oxides including CuO cause adverse effects via the Trojan horse mechanism, i.e. cellular uptake of ENM in particle form, their lysosomal dissolution and subsequent rupture of the lysosome which results in adverse effects such as cell death and induction of inflammation [47–50]. In this study, the dissolution rates of the tested CuO ENM in physiological saline showed that the materials remain in particulate form over 24 h. Furthermore, the minute levels of dissolved copper detected in tested material samples, did not reveal a clear correlation with neutrophil counts in BAL – an endpoint which was clearly an ENM-driven effect. Thus, we conclude that the possible extracellular dissolution of the CuO nanomaterials does not play a major role in the toxic response of the tested materials and the adverse pulmonary effects are more likely triggered by intracellular dissolution.

Suppressive and other beneficial properties of surface PEGylation have been observed earlier and therefore, the modification has found use in nanomedicine on anti-cancer drugs for prolonging their circulation and reducing off-target effects [51, 52]. One of the explanations for the suppressive effects of surface PEGylation of ENM could be that it renders the particles invisible for macrophages [53]. For example, similar surface functionalizations that were studied here have been investigated previously by Rehberg et al. who explored the uptake of pristine and coated quantum dots (QD) in post capillary venules in mouse cremasteric tissue. The authors found that carboxyl- and amine-QD were ingested by perivascular macrophages and/or endothelial cells whereas PEG-QD were seen in-between endothelial cells, associated with amorphous lipid-containing material [54]. It has been previously reported that PEG inhibits cell adhesion non-specifically, not by binding to a cell surface receptor [55]. Thus, it is likely the coating itself, not the functional surface area or the size of the nanomaterial and its aggregates, that hinders the recognition of the PEG-coated particles. However, differences in the uptake of the surface-modified nanomaterials by phagocytes may in turn lead to differences in leucocyte trafficking and recruitment, and consequently, differing immune responses and health outcomes. Therefore, we speculate that surface chemistry is a significant factor and it contributes to the development of the ENM-induced pulmonary effects seen in our study.

To understand how CuO materials modulate OVA-induced AAI, DEG sets of PBS-challenged mice were compared to the ones of OVA-challenged mice by IPA. We evidenced that a sub-cluster of T cell regulation pathways was commonly suppressed at the highest dose regardless of the disease status. With the exception of OVA-challenged mice treated with lower doses of the ENM, administration of CuO materials suppressed cell cycle in G2/M DNA damage checkpoint. It is well known that CuO materials produce ROS that initiate mitochondrial-mediated apoptosis pathway and consequently cause mitochondrial damage, activation of p53 and caspase 3 leading the cell to apoptotic death [56, 57]. It is also reported that metal oxide ENM abrogate cell cycle in G2/M phase which gives the materials a potential to be developed into anticancer agents [58, 59]. In contrast to arrest of G2/M phase transition, other cell cycle pathways were activated by the exposure to CuO materials. Expansion and renewal of cell populations, however, are common processes in inflamed tissues and hence their activation is expected. Exposure to CuO materials activated also several inflammatory pathways, all of whose activation was generally more distinguishable in PBS-challenged mice than in OVA-challenged mice. Lower activation in OVA-challenged mice appears likely due to an already existing inflammation in OVA-challenged control mice against which the ENM-exposed groups were compared with. In addition, we were interested in understanding whether CuO materials regulate inflammation differently depending in PBS- and OVA-challenged groups by comparing activation scores of the canonical pathways. Consequently, majority of the pathways affected by CuO, CuO COOH or CuO NH_3_ exposure had a similar activation direction in both, PBS- and OVA-challenged mice. Overall, these data indicate that the pathways affected by CuO materials do not differ dramatically between healthy and allergic lungs.

## Conclusions

In this study, we evidenced that respiratory exposure to pristine or modified CuO materials does not dramatically influence classical signs of allergic airway inflammation, however, the materials worsen the condition by adding another inflammatory characteristic - pulmonary neutrophilia, to the already existing allergic condition. Global transcriptomic analysis of the lung tissue showed that CuO PEG has significantly lower potential in triggering changes in healthy and asthmatic lungs compared with CuO and its carboxylated and methylaminated derivates. General comparison of the materials at transcriptomics level revealed that CuO, CuO COOH and CuO NH_3_ commonly enriched neutrophil-related biological processes, especially in PBS-challenged mice. Further analysis of the functionalization influence showed that PEGylation of the core CuO suppresses the effects triggered by the pristine particles. Comparison of PBS- and OVA-challenged mice revealed that pathways affected by exposure to CuO materials do not significantly differ in healthy and allergic lungs. Furthermore, we demonstrated that the materials affect T cell regulation, inhibit cell cycle at G2/M phase transition and activate several inflammatory pathways.

Altogether, we found that exposure to core CuO as well as its functionalized derivates exacerbate allergic airway inflammation by causing neutrophilia in the lungs. However, our results also showed that surface PEGylation can be a promising strategy for suppressing the adverse effects of inhaled pristine CuO. The data of this study provide information for health and safety assessment of modified CuO materials. Moreover, the knowledge can be useful in the development of CuO-based nanomedical applications.

## Methods

### Characterization of nanomaterials

The nanomaterials used in the present study have been extensively characterised in ultrapure water and biological media, and for extractable fractions of Cu metal in gut salines [15, 46, 60]. Briefly, the characterisation followed Vassallo et al. with additional measurements in a representative physiological saline (Krebs saline, in mmol l^− 1^: NaCl, 118.6; KCl, 4.7; CaCl_2_, 2.5; MgSO_4_, 1.2; KH_2_PO_4_, 1.2; NaHCO_3_, 25.1; Glucose, 10.0; HEPES, 10.0, and at pH 7.4) [15]. The nanomaterials included the uncoated CuO ENM, and those coated with –NH_4_^+^, −COOH or –PEG respectively. The precise details of how the coatings were synthesised and attached to the ENM core is commercially sensitive information of the suppliers, but for clarity we use the term ‘–NH_4_^+^’ to mean an –NH_3_ terminal ligand that has been ionised with H^+^ ions to achieve positive charge. The CuO ENMs were provided by PlasmaChem GmbH (Berlin, Germany) as part of the Nanosolutions EU project. The characterisation included measurements of the primary particle sizes, the dispersion of the particles in ultrapure water as well as in Krebs physiological saline, zeta potential in ultrapure water, and dialysis experiments to assess any dissolution of dissolved Cu from the particles. The CuO NPs were first examined using transmission electron microscopy (TEM, JEOL-1200EX II) for the primary particle size. Fresh stock suspensions, at 100 mg l^− 1^ nominal concentration, were prepared in Milli-Q water and sub-samples were examined visually with *n* = 60 measurements of particle diameter per sample (conducted manually using ImageJ). The particle size distribution of the ENM in the stock dispersions were also measured by nanoparticle tracking analysis (NTA) using a Nanosight LM 10 (Malvern Instruments, UK). Three sub-samples from each of the fresh stock suspensions were vortexed for 10 s immediately before analysis by NTA (Table 1). Zeta potential of CuO dispersions in ultrapure water (pH 5) was determined by laser Doppler electrophoresis (Zetasizer Nano ZS, Malvern Instruments, UK) as an average of five separate measurements.

Dialysis experiments were conducted in Milli-Q water at room temperature to measure the degree of copper metal ion dissolution from all the ENM. Dialysis bags were filled with 8 ml of the appropriate test suspension at 100 mg l^− 1^ nominal concentration, and suspended in a 600 ml beaker containing 492 ml of Milli-Q water (in triplicate beakers). Samples of 1 ml were taken from the external compartment of the beaker at time zero, 30 min, 1, 2, 3, 4, 6, 8 or 12 and 24 h for total Cu determination by ICP-OES or ICP-MS. The data were subsequently fitted to a rectangular hyperbola (using SigmaPlot 13), and the maximum initial dissolution rate calculated from the maximum slope.

### Total copper concentrations

The total copper concentration in the samples of the original nanopowders were determined following *aqua regia* digestion. In the case of water or saline samples from the dialysis experiments, these were diluted in nitric acid. Total Cu concentrations were determined by inductively coupled plasma optical emission spectrophotometry (ICP-OES, Thermo Scientific, iCAP 7000 Series), or equivalent mass spectrophotometry (ICP-MS, Thermo Scientific, X Series 2) as appropriate. The instrument detection limit for the ICP-OES was 0.008 mg l^− 1^ Cu and for the ICP-MS was 0.003 μg l^− 1^ Cu. Briefly, samples were acidified, matrix-matched to the ICP-OES/ICP-MS standard metal solutions used for calibration, with 0.8 mg l^− 1^ yttrium as an internal standard. Sample blanks were included every 10 samples in each run of the instruments.

### Dispersion preparation

CuO, CuO COOH and CuO NH_3_ were provided as powders and CuO PEG as an aqueous suspension. At the time of the study, it was not possible to calculate equivalent molar doses of Cu because of uncertainty of the stoichiometry and the technical challenges in measuring the mass attributed to the coating. Thus, equivalent mass of the whole material was used for dosing [46]. Stock dispersions were prepared similarly as described earlier [61]. Briefly, CuO powders were dispersed in endotoxin-free water (HyClone Laboratories, Inc., Logan, UT) to a stock suspension of 1 mg/ml. The suspensions were sonicated for 5 min by probe sonicator (Branson Sonifier 250, Branson Ultrasonic Co., Danbury, CT, USA) using a power output corresponding to a delivered power of 13 W. 6.5% aqueous suspension of CuO PEG was diluted 10x in endotoxin-free water to obtain a stock dispersion of 6.5 mg/ml. Stock dispersion was sonicated for 2 min. Further dilutions of 50, 200 and 800 μg/ml (2.5, 10 and 40 μg/mouse, respectively) were prepared in Dulbecco’s PBS (Gibco, Life Technologies, Carlsbad, CA) with or without ovalbumin (OVA; Sigma-Aldrich Co, St. Luis, MO) shortly after sonication and used immediately for orophagyngeal aspirations.

### Mice and sensitization

Female BALB/c mice (aged 6–8 weeks) were obtained from Scanbur A/S (Karlslunde, Denmark) and quarantined for 1 week. The mice were housed in groups of four in transparent plastic cages bedded with aspen chip and were provided standard mouse chow diet (Altromin no. 1314 FORTI, Altromin Spezialfutter GmbH & Co., Germany) and tap water ad libitum when not being treated. The environment of the animal room was carefully controlled, with a 12 h dark-light cycle, temperature of 20–21 °C, and relative humidity of 40–45%. The experiments were performed in agreement with the European Convention for the Protection of Vertebrate Animals Used for Experimental and Other Scientific Purposes (Strasbourg March 18, 1986, adopted in Finland May 31, 1990). All experiments were approved by the State Provincial Office of Southern Finland (ESAVI-3241-04.10.07-2013, permission number PH701A).

During the sensitization period, each mouse (eight per group) received ip a mixture of 50 μg of OVA and 2 mg of aluminum/magnesium hydroxide (Alum; Imject® Alum, Pierce Biotechnology, Rockford, IL) in 100 μl of Dulbecco’s PBS on day 1 and 10. After 10-day recovery, mice were exposed to 50 μl of Dulbecco’s PBS or 50 μg of OVA in 50 μl of Dulbecco’s PBS with or without dispersed CuO materials via oropharyngeal aspiration in isoflurane anaesthesia (Univentor 400 Anaesthesia Unit, Abbott Laboratories, IL) on four consecutive days. CuO materials were tested at three doses, 2.5, 10 and 40 μg/mouse/administration. Mice were sacrificed using an overdose of inhaled isoflurane 24 h after the last administration (Additional file 5: Figure S1).

### Sample collection

Blood was collected from the vena cava (hepatic vein), and the lungs were lavaged with Dulbecco’s PBS (800 μl for 10 s) via the tracheal tube. The mouse chest was opened, and half of the left pulmonary lobe was removed, divided in two, stabilized in RNAlater® solution (Life Technologies Ltd., Paisley, UK) and stored at − 70 °C for total RNA isolation and microarray analysis. A part of the left lobe was embedded in Tissue-Tek® O.C.T.™ compound (Sakura Finetek, Alphen aan Den Rijn, The Netherlands), quick-frozen and stored at − 70 °C for immunohistochemical stainings. The rest of the lungs were formalin-fixed, embedded in paraffin, cut, affixed on slides, and stained with hematoxylin and eosin (H&E) and periodic acid-Schiff (PAS) solutions.

### BAL cell counts

100 μl of BAL samples were cytocentrifuged on slides at 1000 rpm for 10 min (Miles Scientific Cyto-Tek centrifuge, Sakura Finetek). The slides were air-dried and stained with May Grünwald-Giemsa (MGG). BAL cell differentials (number of macrophages, neutrophils, eosinophils and lymphocytes) were obtained as average counts from three high-power fields (HPF). The cells were counted at 50x under light microscopy (Leica DM 4000B; Leica, Wetzlar, Germany).

### Lung histology

Recruitment of inflammatory cells into the lungs and morphological alterations of the tissue were assessed after H&E staining. The number of mucus-producing goblet cells was determined after PAS-staining from three bronchi per mouse in control group and six bronchi per mouse in treatment groups by counting PAS+ cells from 200 μm of bronchus surface under light microscope.

Quantification of histological alterations was performed by measuring all inflammatory areas and areas including nuclear dust of one lung section per slide at a total magnification of 100x with AxioVision V4.8.2 software (Zeiss, Oberkochen, Germany). The results were expressed as averages of the measured areas.

### Immunohistochemistry

Immunoperoxidase staining was used to detect CD3+, CD4+ and CD8+ lymphocytes in the lung tissue. Briefly, 4-μm frozen sections were fixed with cold acetone and stained with anti-mouse CD3 antibody (Ab; clone 17A2), anti-mouse CD4 Ab (clone RM4–5) and anti-mouse CD8 Ab (clone 53–6.7). All primary Abs were purchased from BD Biosciences Pharmingen (San Diego, California). Biotin-conjugated secondary Ab anti-rat IgG (H + L) was purchased from Vector Laboratories. Number of immunohistochemically stained positive cells was counted under light microscopy at × 400 magnification from slides of five samples/group as an average of three HPF/slide.

### mRNA expression of cytokines in lung tissue

Lung samples were homogenized in 1 ml of TRIsure reagent (Bioline Reagents Ltd., London, UK) in Lysing matrix D tubes (MP Biomedicals, Illkirch, France) with a FastPrep FP120 machine (BIO 101, Thermo Savant, Waltham, MA, USA). The RNA extraction was performed following instructions provided by Bioline Reagents. The quantity and purity of isolated RNA was determined by NanoDrop spectrophotometer (ND-1000, Thermo Fisher Scientific Inc., Wilmington, NC, USA). Complementary DNA (cDNA) was synthesized from 500 ng of total RNA in a 25 μl reaction using MultiScribe Reverse Transcriptase and random primers (The High-Capacity cDNA Archive Kit, Applied Biosystems, Foster City, CA, USA) according to the manufacturer’s protocol. The synthesis was performed in a 2720 Thermal Cycler (Applied Biosystems, Carlsbad, CA, USA) starting at 25 °C for 10 min and continuing at 37 °C for 120 min. Primers and probes (18S ribosomal RNA, TNF, IL-33, IL-13, CCL2, CCL7, CCL11) for PCR analysis were ordered as pre-developed assay reagents from Applied Biosystems. The PCR assays were performed in 96-well optical reaction plates with Relative Quantification 7500 Fast System (7500 Fast Real-Time PCR system, Applied Biosystems) by the manufacturer’s instructions. Amplifications were done in 11 μl reaction volume containing TaqMan universal PCR master mix and primers provided by Applied Biosystems and 1 μl of cDNA sample. Ribosomal 18S was used as an endogenous control.

### Cytokine levels in BAL supernatants

Protein secretion of IL-13 and CCL2 was measured in BAL supernatants by commercial mouse uncoated ELISA kits (Invitrogen, San Diego, CA). The assays were performed according to the manufacturer’s instructions and ELISA plate reader (Multiscan MS, Labsystems, Finland) was used to record the absorbances.

### DNA microarrays and statistical analysis of the data

Total RNA samples isolated from lung tissue as described above were quantified and quality checked by NanoDrop and Agilent Bioanalyzer 2100 (Agilent Technologies, Santa Clara, CA, USA), respectively. Samples with RNA integrity number (RIN) > 8 were selected for the analysis. Independent pools of two RNA samples (total of 100 ng) were used to synthesize cDNA which was transcribed to cRNA using T7 RNA polymerase amplification method (Low Input Quick Amp Labeling Kit, Agilent Technologies), according to the instructions of the manufacturer. cRNAs were labeled with Cy3 and Cy5 dyes (Agilent Technologies) and thereafter cleaned up by using Qiagen’s RNeasy mini spin columns (Qiagen, GmbH, Hilden, Germany). 300 ng of a Cy3-labeled sample and 300 ng of corresponding Cy5-labeled sample were combined (total 600 ng). cRNA samples were fragmented and hybridized to the Agilent 2-color 60-mer oligo arrays (Agilent SurePrint G3 Mouse Gene Expression v2 GE 8x60K). The slides were washed and scanned with Agilent Microarray Scanner G2505C (Agilent Technologies) and the raw intensity values were obtained with the Feature Extraction software, version 11.0.1.1 (Agilent Technologies). Raw data was quality checked according to the Agilent standard procedures.

The median foreground intensities were imported into the R software [62] and analyzed with limma package within the BioConductor. Briefly, log2 transformation and quantile normalization was performed. Subsequently, batch effects derived from the labeling and array-specific variance were removed using the ComBat method [63] implemented in the sva package [64, 65]. The same batches were taken also into account when limma package [66] was used. The values of the probes recognizing the same NCBI Entrez Gene identifiers were further averaged into the final expression matrix. The microarray data is deposited in NCBI Gene Expression Omnibus (GEO) database [67] and are accessible through GEO Series accession number GSE122197 (https://www.ncbi.nlm.nih.gov/geo/query/acc.cgi?acc=GSE122197). Differentially expressed genes were identified by using linear models and empirical Bayes pairwise comparisons (post hoc adjusted *P* < 0.05 and linear fold change (FC) > |1.5|). The resulting gene sets after Benjamini and Hochberg post hoc correction [63] were considered to be significant and were further studied.

### Analysis tools

Enrichment analyses of biological processes were performed using Enrichr [17, 18] and PANTHER [19] tools. REVIGO [68] was used to summarize lists of gene ontology (GO) terms. Canonical Pathway Analyses were carried out through the use of IPA [16]. Unless otherwise specified, the cut-off for a biological process or pathway to be considered significantly enriched was set at adjusted *p* value of 0.05. Bar and line plots were constructed using GraphPad Prism 7 (GraphPad Software Inc., San Diego, CA, USA) or IPA software. Heat maps and 2D scatter plots were created with Perseus [69, 70], Venn diagrams with Venny 2.1.0 [71]. Tile plots were prepared by R software [62] using the codes obtained from REVIGO tool [68].

### Statistical analysis of cell counts and mRNA expression levels

For statistical analysis of cell counts and PCR results, the Analysis of Variance (ANOVA) was performed. Tukey honest significant differences post-hoc testing was carried out after ANOVA for selected pair-wise comparisons. A *P*-value of < 0.05 was considered to be statistically significant. Validation of microarray results by PCR was evaluated with Pearson’s correlation test. The results were considered accurate when Pearson’s r value was ~ 1.

## Additional files

Additional file 1: **Table S1.** Total number of BAL cells and relative portion of differential immune cells. The numbers are presented as mean values ± standard error of mean (SEM). Statistically significant differences of total cell numbers between experimental groups and PBS-challenged control mice are marked with “*” whereas the ones between experimental groups and OVA-challenged control mice are marked with “•”. ***/•••*P* < 0.001. HPF, high power field; OVA, ovalbumin; PBS, phosphate buffered saline. (TIF 1108 kb)

Additional file 2: **Table S2.** Lists of the differentially expressed genes from the microarray experiment. The tables with the differentially expressed genes in each of the relevant pairwise comparisons (each experimental group compared against corresponding PBS-challenged or OVA-challenged controls) are reported in sheets of the Microsoft Excel archive, named accordingly. In each table, the significantly differentially expressed genes (Benjamini and Hochberg post hoc corrected *P* value < 0.05 and absolute log2 fold change > 0.58) are ordered according to the decreasing log2 fold change. The Agilent probe IDs and gene symbols are shown. Additionally, for each significant gene, the average expression across the microarray samples, the t-test value, the nominal *P* value, the adjusted *P* value and the B value are also reported. (XLSX 1842 kb)

Additional file 3: **Table S3** Lists of the differentially expressed genes from the microarray experiment. Experimental groups of PBS-challenged or OVA-challenged mice that were treated with the same CuO material were combined and compared against their corresponding controls. The tables with the differentially expressed genes of each pairwise comparison are reported in sheets of the Microsoft Excel archive, named accordingly. In each table, the significantly differentially expressed genes (Benjamini and Hochberg post hoc corrected *p*-value < 0.05 and absolute log2 fold change > 0.58) are ordered according to the decreasing log2 fold change. The Agilent probe IDs and gene symbols are shown. Additionally, for each significant gene, the average expression across the microarray samples, the t-test value, the nominal *P* value, the adjusted *P* value and the B value are also reported. (XLSX 256 kb)

Additional file 4: **Table S4.** Lists of the differentially expressed genes from the microarray experiment. Experimental groups treated with modified CuO materials were compared against the groups treated with the corresponding dose of core CuO in PBS-challenged or OVA-challenged mice. The tables with the differentially expressed genes of each pairwise comparison are reported in sheets of the Microsoft Excel archive, named accordingly. In each table, the significantly differentially expressed genes (Benjamini and Hochberg post hoc corrected p-value < 0.05 and absolute log2 fold change > 0.58) are ordered according to the decreasing log2 fold change. The Agilent probe IDs and gene symbols are shown. Additionally, for each significant gene, the average expression across the microarray samples, the t-test value, the nominal *P* value, the adjusted *P* value and the B value are also reported. (XLSX 338 kb)

Additional file 5: **Figure S1.** Murine model of allergic airway inflammation. BALB/c mice were sensitized intraperitoneally (ip) with a mixture of ovalbumin and aluminium/magnesium hydroxide (OVA/Alum) on day 1 and 10. After a 10-day recovery period, 2.5, 10 or 40 μg/mouse of CuO nanomaterials dispersed in PBS with or without OVA were administered repeatedly to mice by oropharyngeal aspiration on days 20–23. Mice were sacrificed on day 24 for sample collection of bronchoalveolar lavage fluid (BAL) and lung tissue. ENM, engineered nanomaterial; PBS, phosphate buffered saline. (TIF 444 kb)

Additional file 6: **Figure S2.** Example transmission electron microscopy images of engineered CuO nanoparticles in 100 mg l^− 1^ Milli-Q water showing, (A) uncoated CuO core, (B) CuO-COOH, (C) CuO-NH_4_^+^ and, (D) CuO-PEG NPs. The respective Nanosight graphs show the particle distribution (bin sizes are hydrodynamic diameter) of the nanomaterials in 100 mg l^− 1^ Milli-Q water solutions. From Besinis and Handy, unpublished. (TIF 2186 kb)

Additional file 7: **Figure S3.** Example transmission electron microscopy images of engineered CuO nanoparticles in 100 mg l^− 1^ Krebs physiological saline showing, (A) uncoated CuO core, (B) CuO-COOH, (C) CuO-NH_4_^+^ and, (D) CuO-PEG NPs. The respective Nanosight graphs show the particle distribution (bin sizes are hydrodynamic diameter) of the nanomaterials in 100 mg l^− 1^ Milli-Q water solutions. From Besinis and Handy, unpublished. (TIF 2625 kb)

Additional file 8: **Figure S4.** Dialysis curves showing the release of total dissolved copper from uncoated CuO NPs core, CuO-COOH, CuO-NH_4_^+^ and CuO-PEG NPs over a 24 h period when suspended in (A) Ultrapure Milli-Q water, or (B) Krebs physiological saline. Controls are the respective water or saline without nanomaterials. Data are means ± S.D., *n* = 3 replicates. Curves were fitted using SigmaPlot 13.0 (Systat Software, Inc.) applying the single rectangular two parameter hyperbola equation on the raw data. From Besinis and Handy, unpublished. (TIF 672 kb)

Additional file 9: **Figure S5.** Histological assessment of the lung tissue after exposure to 10 μg/mouse of CuO nanomaterials. BALB/c mice were sensitized ip to OVA/Alum on day 1 and 10, and exposed repeatedly to 10 μg/mouse of CuO nanomaterials dispersed in PBS with or without OVA by oropharyngeal aspiration after a 10-day recovery period. A, H&E-stained lung tissue of PBS- and OVA-challenged mice that were treated with or without CuO nanomaterials. Histological changes were quantified in selected experimental groups and the results were expressed as an average size of inflammatory areas/section (B) and as an average size of areas containing nuclear dust/section (C). Images (A) are shown at × 200 magnification with a 100-μm scale bar. Columns and error bars represent mean values ± standard error of mean (SEM). *P < 0.05; **P < 0.01; ***P < 0.001. (TIF 6184 kb)

Additional file 10: **Figure S6.** Validation of microarray data by PCR and ELISA. On the basis of the DNA microarray results, three genes were selected for validation by PCR taking into account their biological relevance in recruitment of leucocytes or in allergic airway inflammation. PCR results confirmed an upregulation of monocyte-recruiting chemokines CCL2 and CCL7 in lungs of ENM-treated mice compared with respective control mice at both time points, and induction of and eosinophil-attracting chemokine CCL11 in OVA-challenged mice. Microarray measurements were considered valid as the expression measured by PCR was concordant with microarray data and the correlation between the results was significant. In addition, CCL2 was measured at protein level in BAL supernatants by ELISA and the results were highly similar to those obtained by microarray and PCR. Left-sided plots present microarray results, right-sided plots in the top panel and the middle plot in the bottom panel express PCR results. Protein concentrations of CCL2 are presented in the bottom right. Data points and error bars represent mean values ± standard error of mean (SEM). AU, arbitrary unit of fluorescence intensity; RU, relative expression unit. (TIF 1265 kb)

Additional file 11: **Figure S7.** Biomarkers associated to allergic airway inflammation were found up-regulated in ovalbumin (OVA)-challenged mice. Several markers known to play a role in asthma were chosen (pro-allergic IL-33, Th2 type cytokine IL-4, eosinophil-attracting chemokines CCL11 and CCL24, Th2 lymphocyte-attracting chemokine CCL17, and mucins MUC5AC and MUC5B), and their microarray gene expression data were compared in OVA-challenged and PBS-challenged mice. All selected markers were significantly up-regulated in mice challenged with OVA indicating that allergic airway inflammation developed in OVA-induced mice. AU, arbitrary unit of fluorescence intensity; PBS, phosphate buffered saline. (TIF 455 kb)

Additional file 12: **Figure S8.** General profiling of canonical pathways and biological processes in OVA-challenged mice after CuO, CuO COOH and CuO NH_3_ exposure. Experimental groups of exposed OVA-challenged mice were merged based on the test material, and their transcriptomic profiles were compared against the one of OVA-challenged controls. A, Number of differentially expressed genes (DEGs; linear fold change (FC) > |1.5|, adjusted *P* value < 0.05) in lungs of OVA-challenged mice exposed to CuO nanomaterials by oropharyngeal aspiration (2.5, 10 and 40 μg/mouse). B, Number of significantly enriched biological processes (adjusted *P* value < 0.05) obtained from analyses of DEG sets shown in (A), and biological process enriched commonly by DEGs of CuO, CuO COOH and CuO NH_3_ exposure with adjusted *P* values. (TIF 848 kb)

Additional file 13: **Figure S9.** DEGs with negative fold change in PBS mice exposed to 2.5 μg of CuO PEG versus core CuO at the same dose belong to processes related to inflammatory response. Treemap of GO biological processes significantly enriched by down-regulated genes in PBS-challenged mice exposed to 2.5 μg of CuO PEG compared against core CuO at the same dose. The size of each rectangle reflects the false discovery rate (FDR) of the corresponding GO term. GO terms are grouped by semantic similarity (SimRel, similarity = 0.5) into the same upper hierarchy term, visualized with different colours. (TIF 1139 kb)

Additional file 14: **Figure S10.** GO biological processes enriched by DEGs in PBS mice exposed to 40 μg of CuO PEG versus core CuO at the same dose belong to processes related to inflammatory response. Treemap of GO biological processes significantly enriched by DEGs with A) a positive fold change and B) a negative fold change. The size of each rectangle reflects the FDR of the corresponding GO term. GO terms are grouped by semantic similarity (SimRel, similarity = 0.5) into the same upper hierarchy term, visualized with different colours. (TIF 1880 kb)

Additional file 15: **Figure S11.** DEGs with a positive fold change in OVA-challenged mice exposed to 40 μg of CuO PEG versus core CuO at the same dose belong to processes related to inflammatory response. Treemap of GO biological processes significantly enriched by up-regulated genes in OVA-challenged mice exposed to 40 μg of CuO PEG compared against core CuO at the same dose. The size of each rectangle reflects FDR of the corresponding GO term. GO terms are grouped by semantic similarity (SimRel, similarity = 0.5) into the same upper hierarchy term, visualized with different colours. (TIF 732 kb)

Additional file 16: **Figure S12.** Number of DEGs and canonical pathways involved in the inflammatory response to CuO materials in PBS-challenged and OVA-challenged mice. A differential expression analysis was performed in which each experimental group was compared against their corresponding negative controls. Thereafter, the activation z-scores of the pathways obtained from IPA were compared in PBS-challenged and OVA-challenged mice. A, A table of DEG numbers specific to PBS- and OVA-challenged groups, and an overlap between them (number of common DEGs). B, A heatmap of (in)activation of predicted canonical pathways with IPA cut-offs of absolute z-score > 2 and -log(*P* value) > 2. (TIF 1300 kb)

Additional file 17: **Figure S13.** Canonical pathways involved in the inflammatory response to CuO materials. A differential expression analysis was performed in which each experimental group was compared against their respective PBS- or OVA-challenged control group. Activation z-scores of the predicted pathways with cut-offs of absolute z-score > 2 and -log(*P* value) > 2 were obtained from IPA and compared in PBS-challenged and OVA-challenged mice. A-D, 2D-scatterplots of canonical pathways affected by A) CuO, B) CuO COOH, C) CuO NH_3_ and D) CuO PEG exposure (40 μg/mouse) in PBS- and OVA-challenged mice. Top 5 pathways of each section with the highest z-scores have been denoted above or below the plot, and marked on the plot with a filled circle. Blue circles refer to correlated and red to anti-correlated pathways. Black circles indicate the pathways that had a z-score of zero in one of the data sets. (TIF 1108 kb)

## Data Availability

The data supporting the conclusions of this article are included within the article and its additional files. The microarray data are available in the NCBI GEO database and are accessible through GEO Series accession number GSE122197 (https://www.ncbi.nlm.nih.gov/geo/query/acc.cgi?acc=GSE122197).
