# Supplementary figures and images for: Surface PEGylation suppresses pulmonary effects of CuO in allergen-induced lung inflammation
Source: Part Fibre Toxicol. 2019 Jul 5;16:28. doi: 10.1186/s12989-019-0309-1 (PMC6612204; doi:10.1186/s12989-019-0309-1)

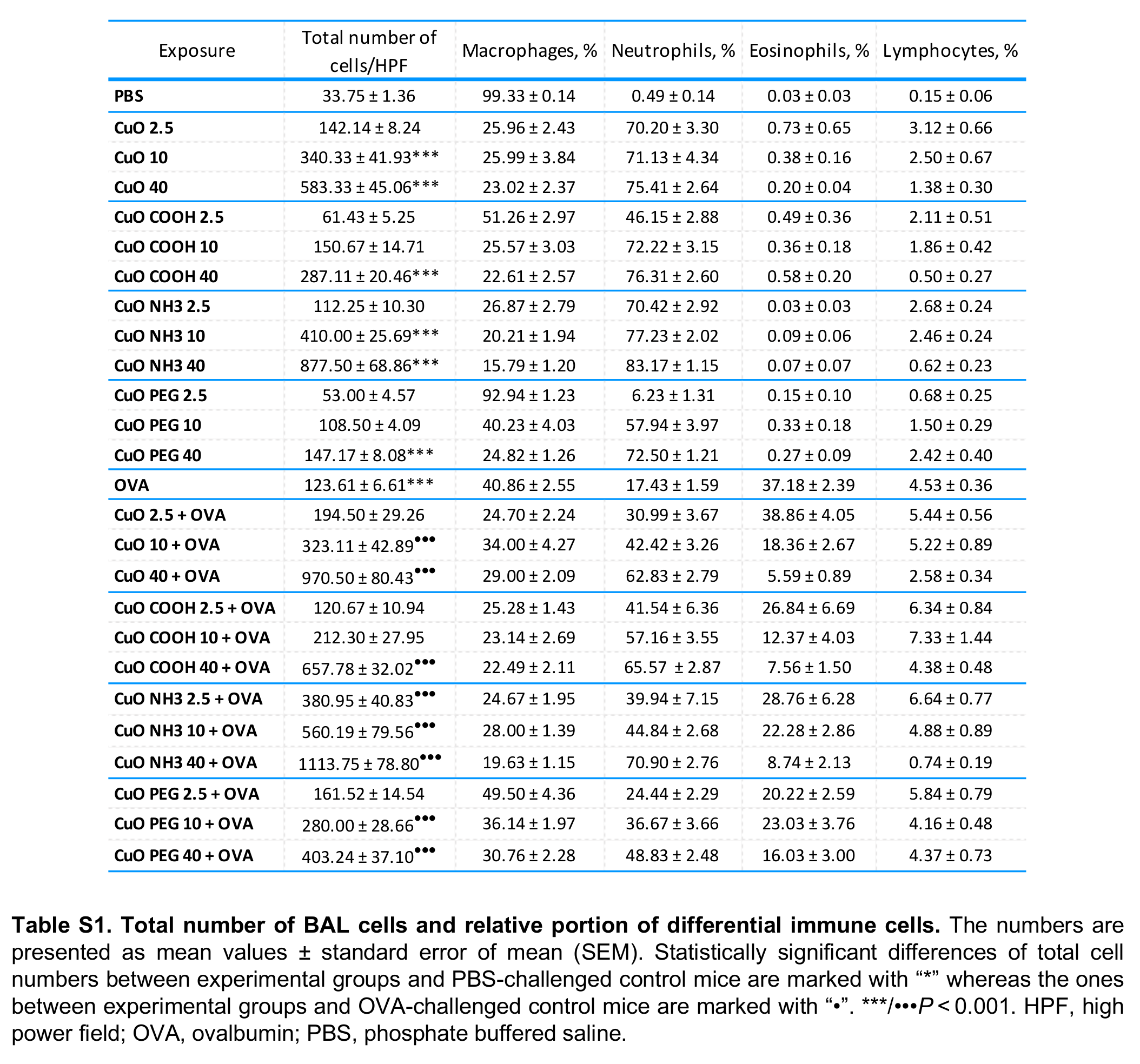

Supplement: Supplementary file 1 — Table S1. Total number of BAL cells and relative portion of differential immune cells. The numbers are presented as mean values ± standard error of mean (SEM). Statistically significant differences of total cell numbers between experimental groups and PBS-challenged control mice are marked with “*” whereas the ones between experimental groups and OVA-challenged control mice are marked with “•”. ***/•••P < 0.001. HPF, high power field; OVA, ovalbumin; PBS, phosphate buffered saline. (TIF 1108 kb) [file 12989_2019_309_MOESM1_ESM.tif]

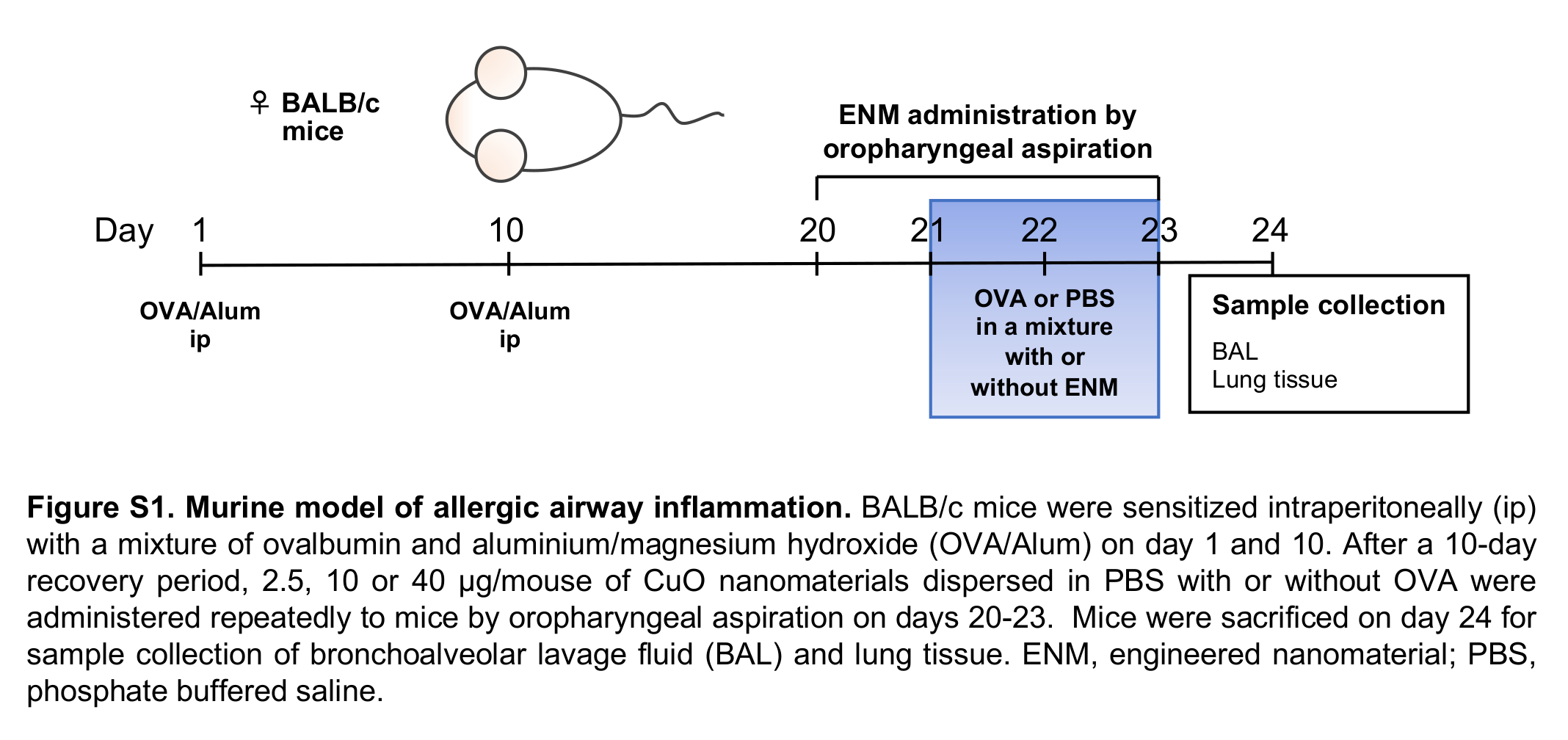

Supplement: Supplementary file 5 — Figure S1. Murine model of allergic airway inflammation. BALB/c mice were sensitized intraperitoneally (ip) with a mixture of ovalbumin and aluminium/magnesium hydroxide (OVA/Alum) on day 1 and 10. After a 10-day recovery period, 2.5, 10 or 40 μg/mouse of CuO nanomaterials dispersed in PBS with or without OVA were administered repeatedly to mice by oropharyngeal aspiration on days 20–23. Mice were sacrificed on day 24 for sample collection of bronchoalveolar lavage fluid (BAL) and lung tissue. ENM, engineered nanomaterial; PBS, phosphate buffered saline. (TIF 444 kb) [file 12989_2019_309_MOESM5_ESM.tif]

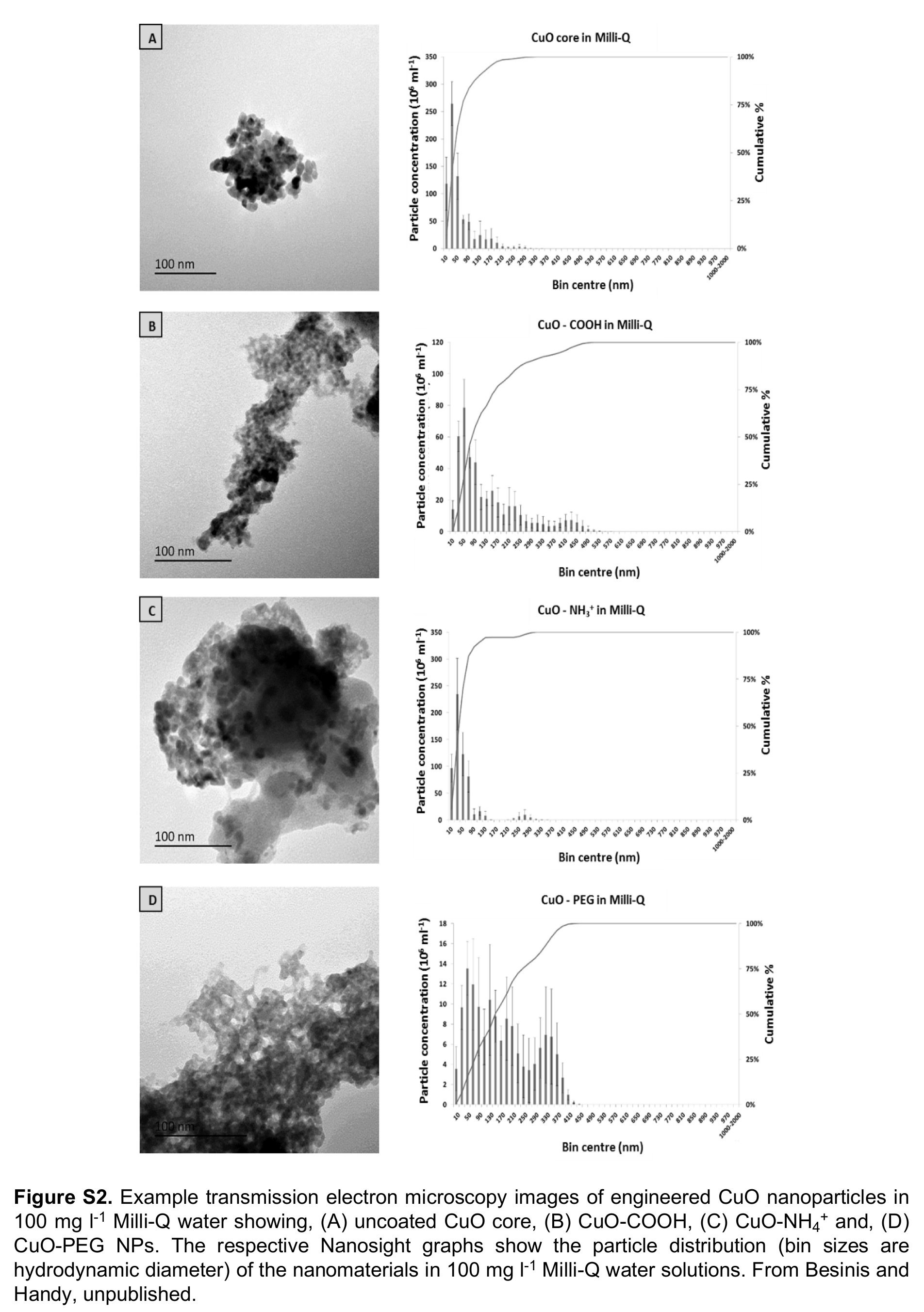

Supplement: Supplementary file 6 — Figure S2. Example transmission electron microscopy images of engineered CuO nanoparticles in 100 mg l− 1 Milli-Q water showing, (A) uncoated CuO core, (B) CuO-COOH, (C) CuO-NH4+ and, (D) CuO-PEG NPs. The respective Nanosight graphs show the particle distribution (bin sizes are hydrodynamic diameter) of the nanomaterials in 100 mg l− 1 Milli-Q water solutions. From Besinis and Handy, unpublished. (TIF 2186 kb) [file 12989_2019_309_MOESM6_ESM.tif]

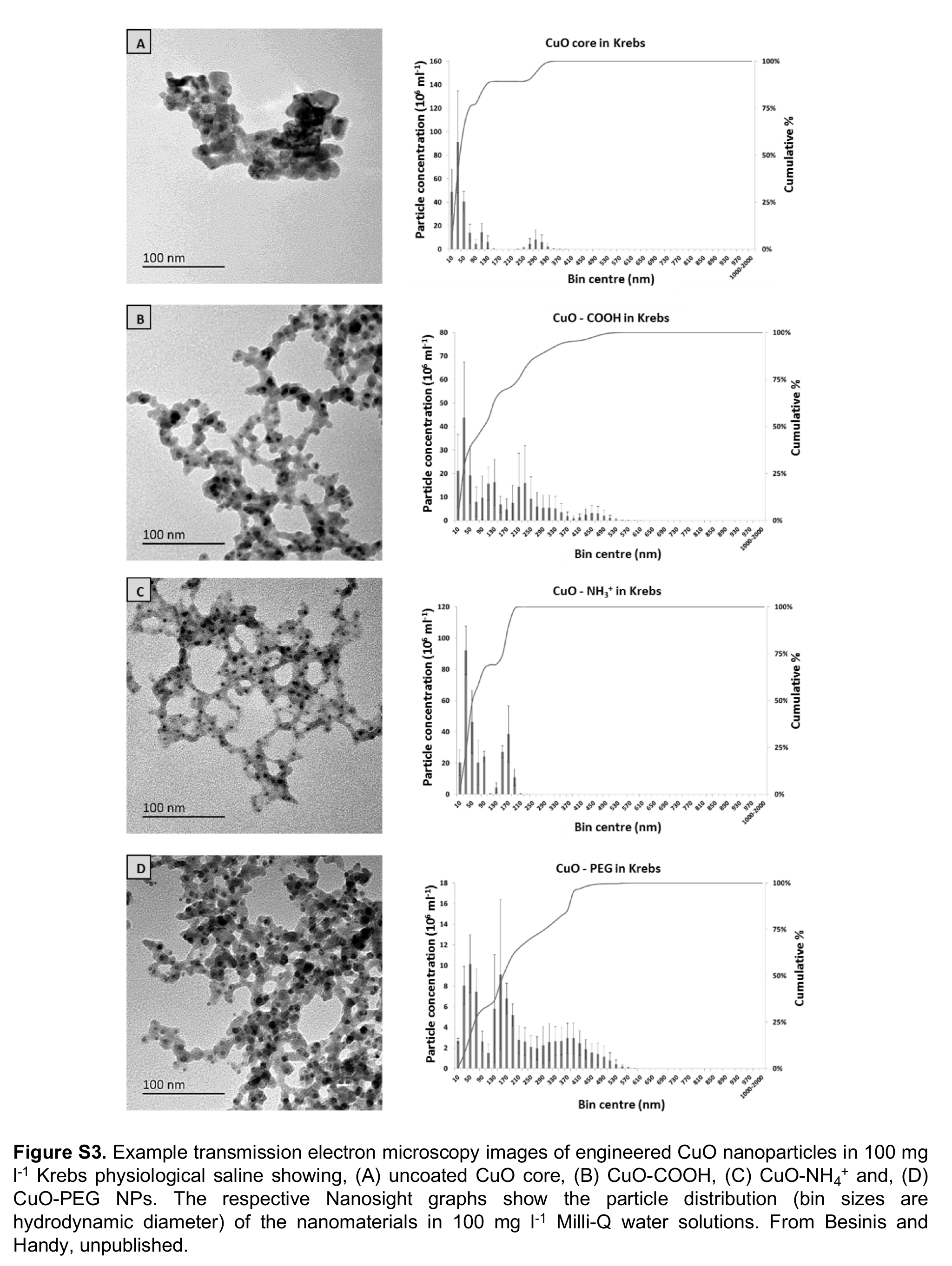

Supplement: Supplementary file 7 — Figure S3. Example transmission electron microscopy images of engineered CuO nanoparticles in 100 mg l− 1 Krebs physiological saline showing, (A) uncoated CuO core, (B) CuO-COOH, (C) CuO-NH4+ and, (D) CuO-PEG NPs. The respective Nanosight graphs show the particle distribution (bin sizes are hydrodynamic diameter) of the nanomaterials in 100 mg l− 1 Milli-Q water solutions. From Besinis and Handy, unpublished. (TIF 2625 kb) [file 12989_2019_309_MOESM7_ESM.tif]

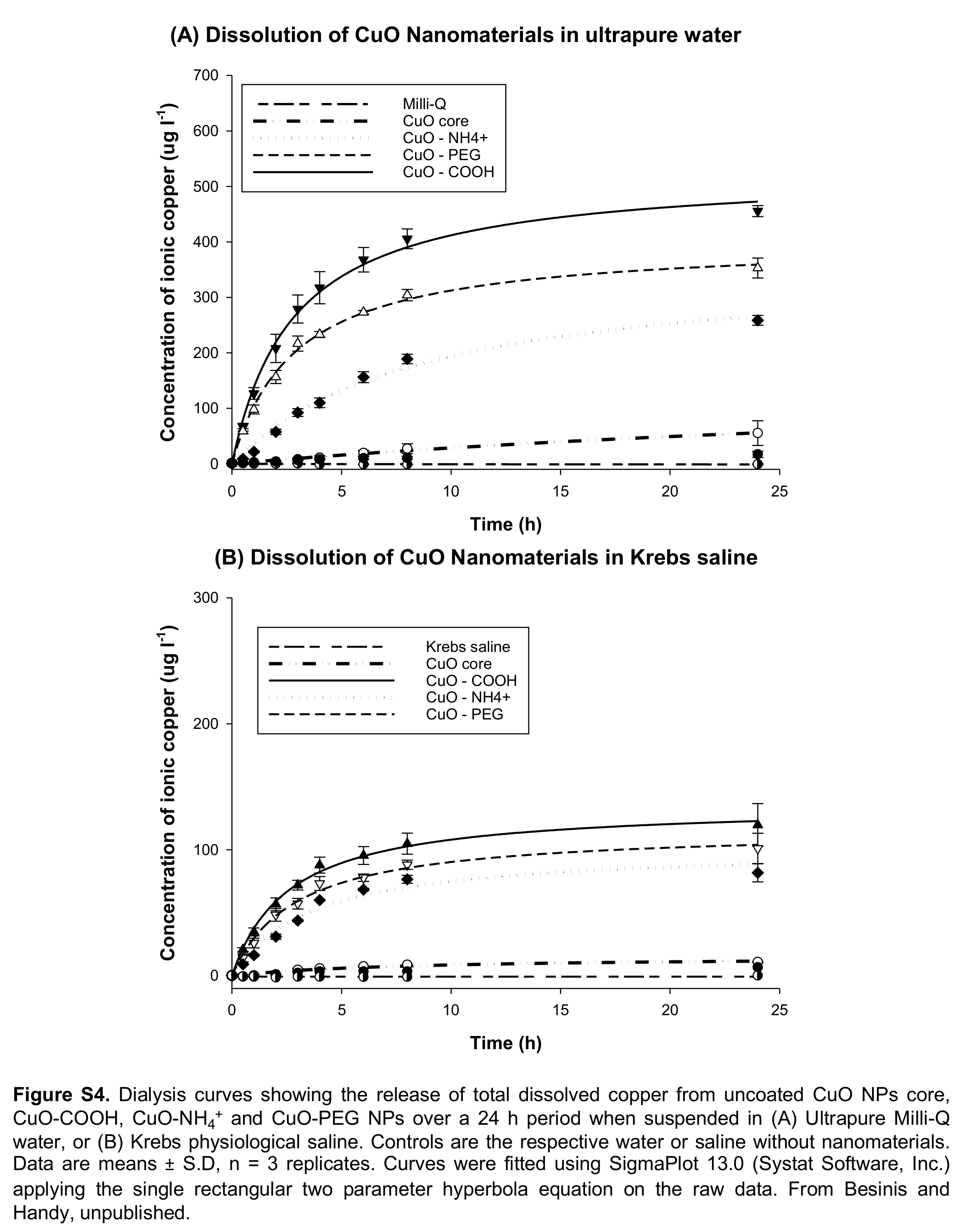

Supplement: Supplementary file 8 — Figure S4. Dialysis curves showing the release of total dissolved copper from uncoated CuO NPs core, CuO-COOH, CuO-NH4+ and CuO-PEG NPs over a 24 h period when suspended in (A) Ultrapure Milli-Q water, or (B) Krebs physiological saline. Controls are the respective water or saline without nanomaterials. Data are means ± S.D., n = 3 replicates. Curves were fitted using SigmaPlot 13.0 (Systat Software, Inc.) applying the single rectangular two parameter hyperbola equation on the raw data. From Besinis and Handy, unpublished. (TIF 672 kb) [file 12989_2019_309_MOESM8_ESM.tif]

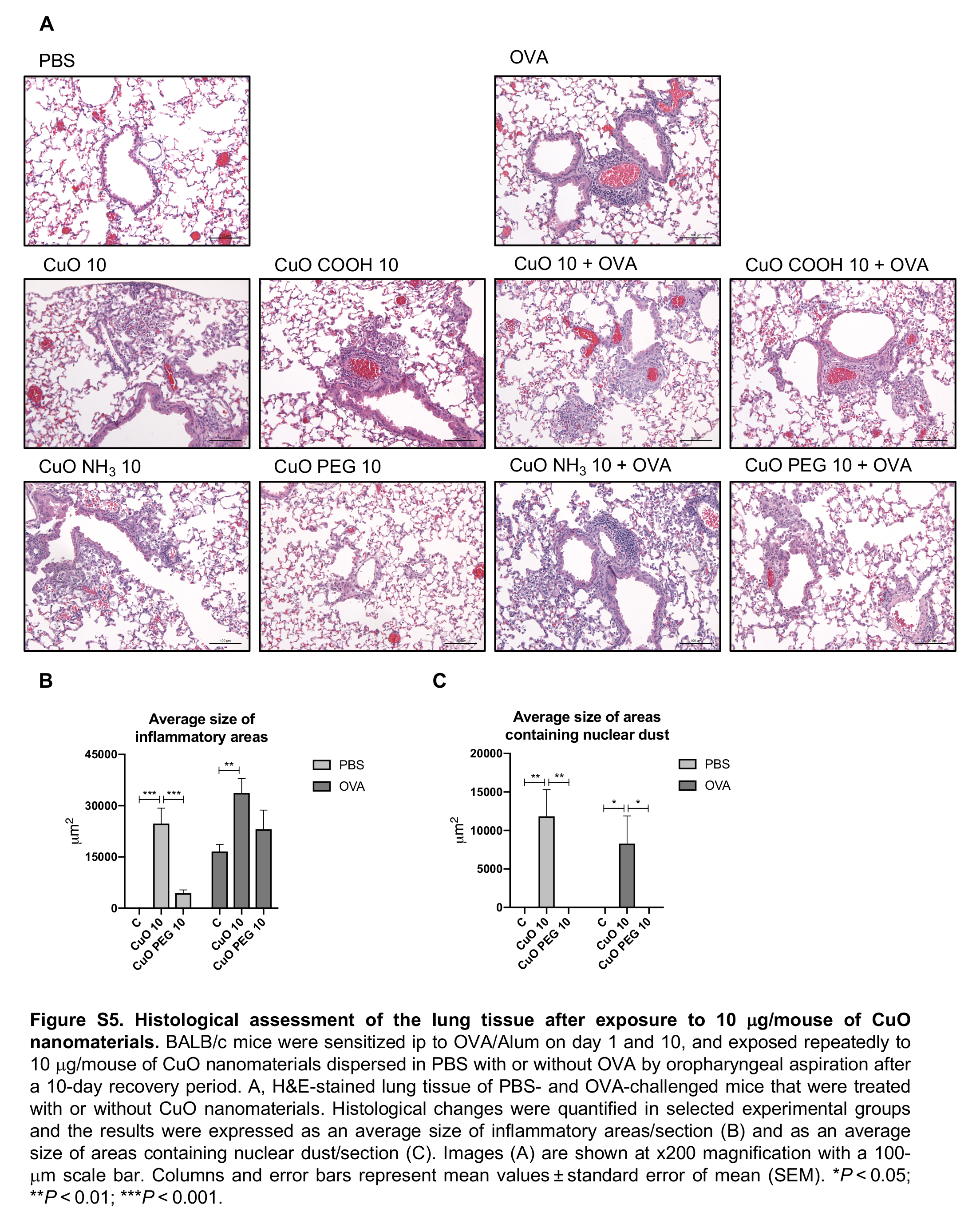

Supplement: Supplementary file 9 — Figure S5. Histological assessment of the lung tissue after exposure to 10 μg/mouse of CuO nanomaterials. BALB/c mice were sensitized ip to OVA/Alum on day 1 and 10, and exposed repeatedly to 10 μg/mouse of CuO nanomaterials dispersed in PBS with or without OVA by oropharyngeal aspiration after a 10-day recovery period. A, H&E-stained lung tissue of PBS- and OVA-challenged mice that were treated with or without CuO nanomaterials. Histological changes were quantified in selected experimental groups and the results were expressed as an average size of inflammatory areas/section (B) and as an average size of areas containing nuclear dust/section (C). Images (A) are shown at × 200 magnification with a 100-μm scale bar. Columns and error bars represent mean values ± standard error of mean (SEM). *P < 0.05; **P < 0.01; ***P < 0.001. (TIF 6184 kb) [file 12989_2019_309_MOESM9_ESM.tif]

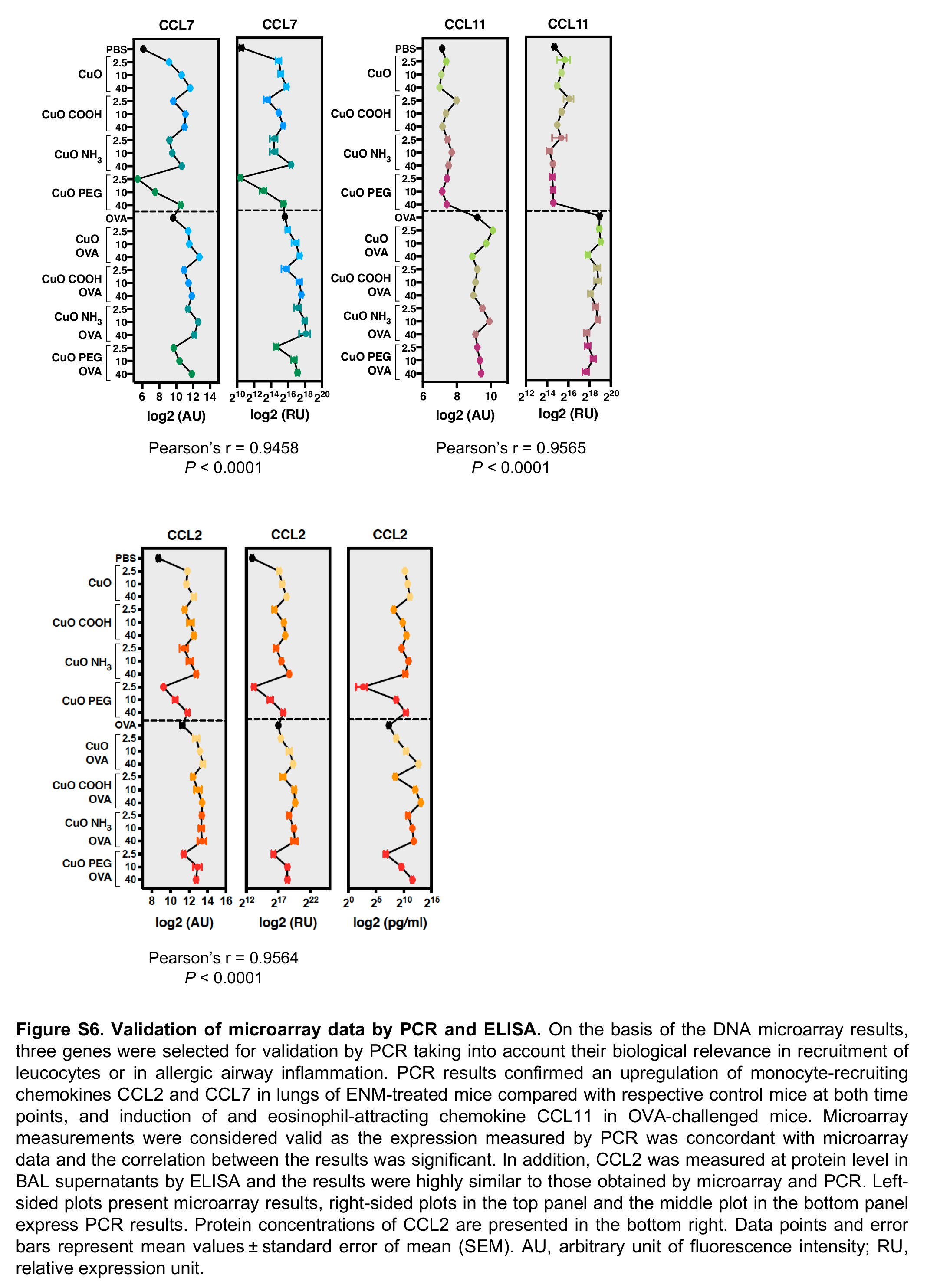

Supplement: Supplementary file 10 — Figure S6. Validation of microarray data by PCR and ELISA. On the basis of the DNA microarray results, three genes were selected for validation by PCR taking into account their biological relevance in recruitment of leucocytes or in allergic airway inflammation. PCR results confirmed an upregulation of monocyte-recruiting chemokines CCL2 and CCL7 in lungs of ENM-treated mice compared with respective control mice at both time points, and induction of and eosinophil-attracting chemokine CCL11 in OVA-challenged mice. Microarray measurements were considered valid as the expression measured by PCR was concordant with microarray data and the correlation between the results was significant. In addition, CCL2 was measured at protein level in BAL supernatants by ELISA and the results were highly similar to those obtained by microarray and PCR. Left-sided plots present microarray results, right-sided plots in the top panel and the middle plot in the bottom panel express PCR results. Protein concentrations of CCL2 are presented in the bottom right. Data points and error bars represent mean values ± standard error of mean (SEM). AU, arbitrary unit of fluorescence intensity; RU, relative expression unit. (TIF 1265 kb) [file 12989_2019_309_MOESM10_ESM.tif]

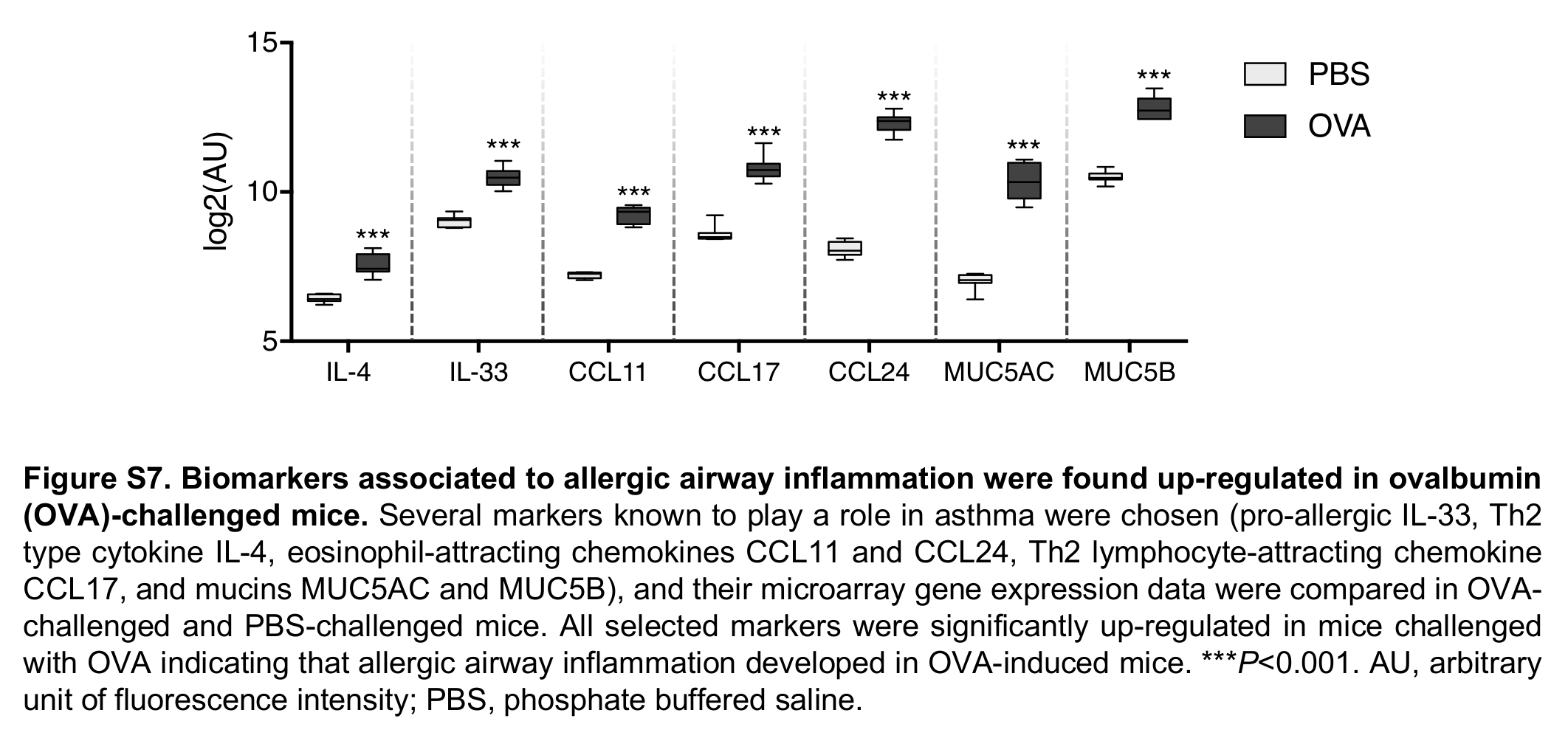

Supplement: Supplementary file 11 — Figure S7. Biomarkers associated to allergic airway inflammation were found up-regulated in ovalbumin (OVA)-challenged mice. Several markers known to play a role in asthma were chosen (pro-allergic IL-33, Th2 type cytokine IL-4, eosinophil-attracting chemokines CCL11 and CCL24, Th2 lymphocyte-attracting chemokine CCL17, and mucins MUC5AC and MUC5B), and their microarray gene expression data were compared in OVA-challenged and PBS-challenged mice. All selected markers were significantly up-regulated in mice challenged with OVA indicating that allergic airway inflammation developed in OVA-induced mice. AU, arbitrary unit of fluorescence intensity; PBS, phosphate buffered saline. (TIF 455 kb) [file 12989_2019_309_MOESM11_ESM.tif]

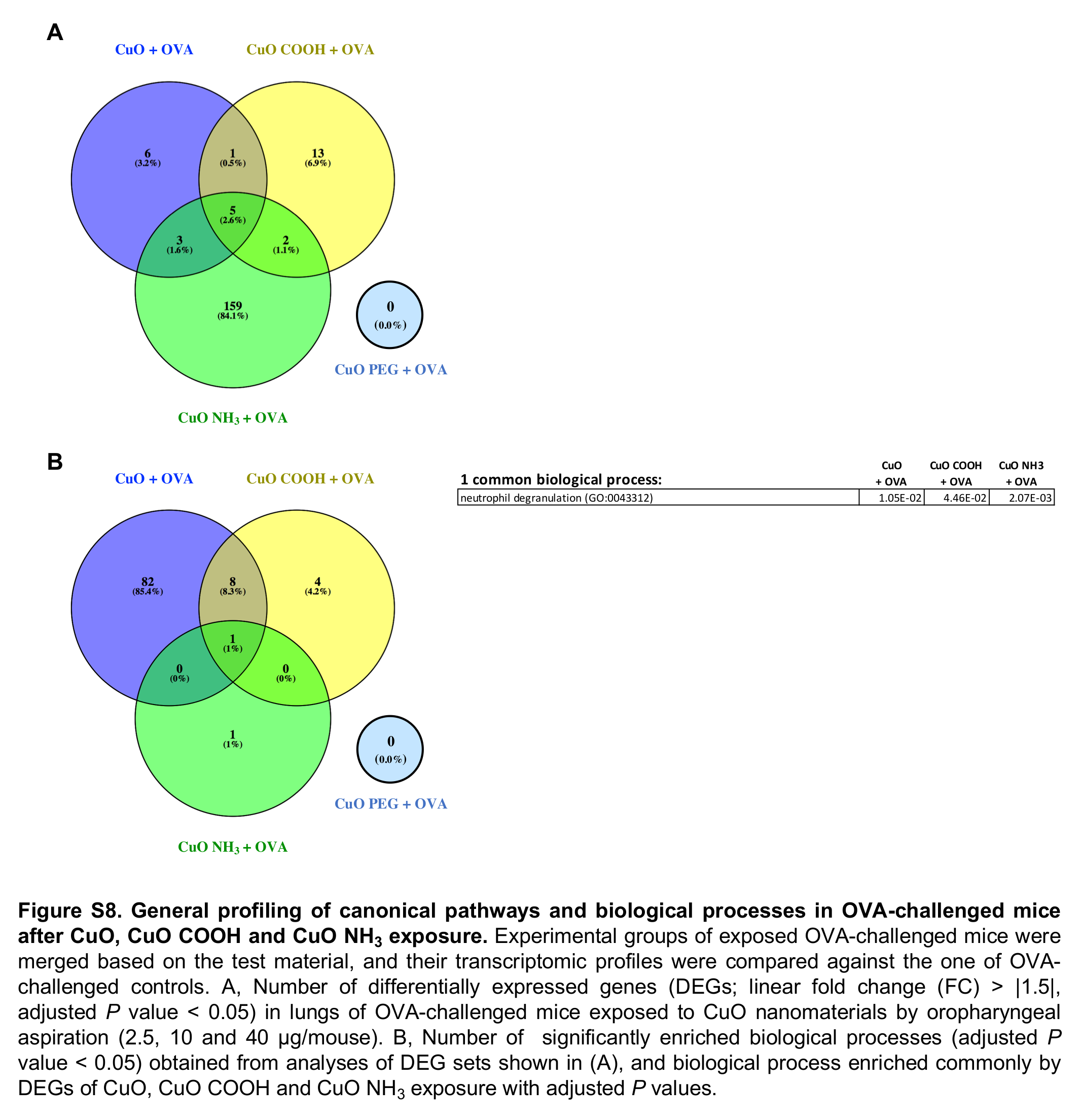

Supplement: Supplementary file 12 — Figure S8. General profiling of canonical pathways and biological processes in OVA-challenged mice after CuO, CuO COOH and CuO NH3 exposure. Experimental groups of exposed OVA-challenged mice were merged based on the test material, and their transcriptomic profiles were compared against the one of OVA-challenged controls. A, Number of differentially expressed genes (DEGs; linear fold change (FC) > |1.5|, adjusted P value < 0.05) in lungs of OVA-challenged mice exposed to CuO nanomaterials by oropharyngeal aspiration (2.5, 10 and 40 μg/mouse). B, Number of significantly enriched biological processes (adjusted P value < 0.05) obtained from analyses of DEG sets shown in (A), and biological process enriched commonly by DEGs of CuO, CuO COOH and CuO NH3 exposure with adjusted P values. (TIF 848 kb) [file 12989_2019_309_MOESM12_ESM.tif]

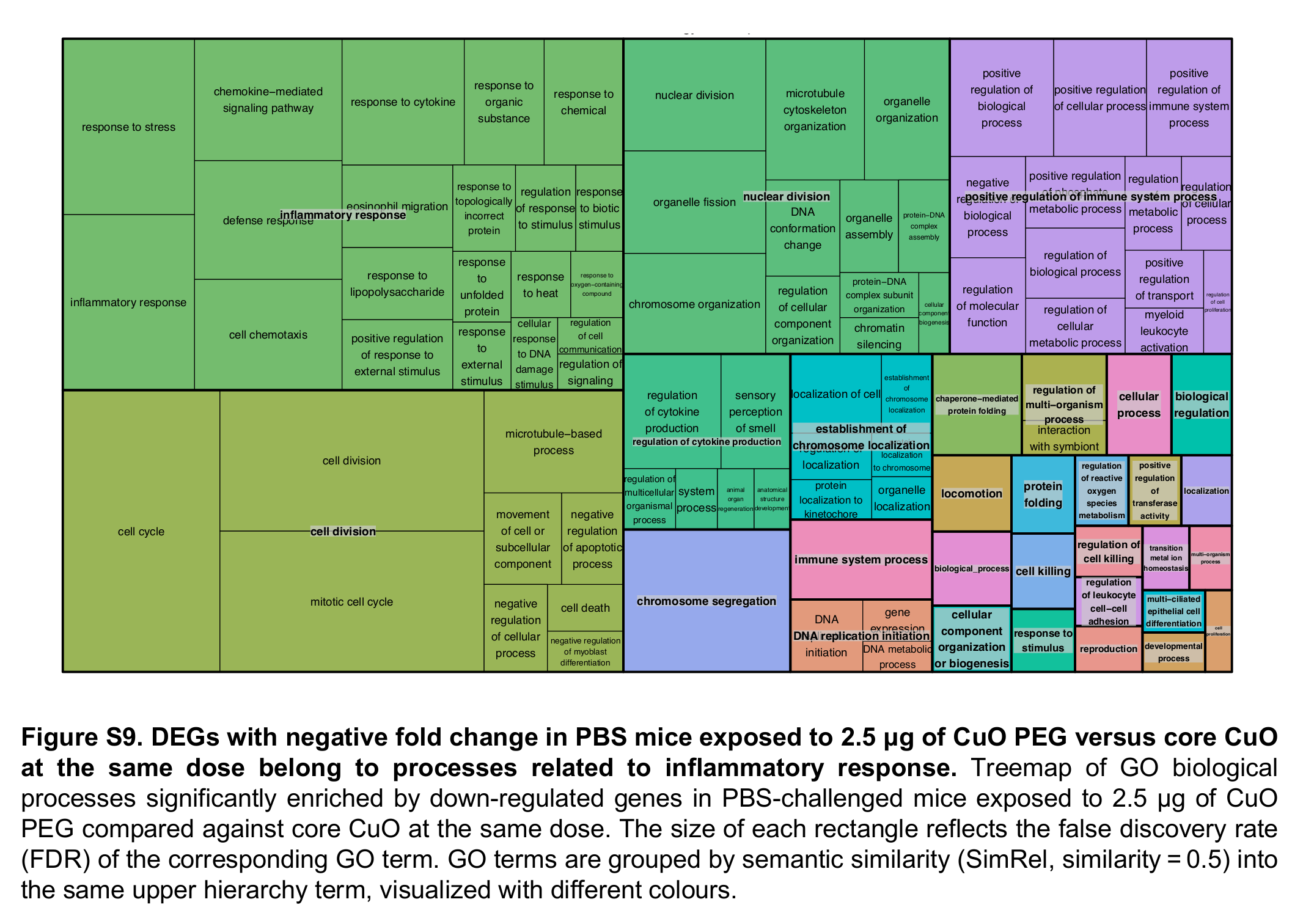

Supplement: Supplementary file 13 — Figure S9. DEGs with negative fold change in PBS mice exposed to 2.5 μg of CuO PEG versus core CuO at the same dose belong to processes related to inflammatory response. Treemap of GO biological processes significantly enriched by down-regulated genes in PBS-challenged mice exposed to 2.5 μg of CuO PEG compared against core CuO at the same dose. The size of each rectangle reflects the false discovery rate (FDR) of the corresponding GO term. GO terms are grouped by semantic similarity (SimRel, similarity = 0.5) into the same upper hierarchy term, visualized with different colours. (TIF 1139 kb) [file 12989_2019_309_MOESM13_ESM.tif]

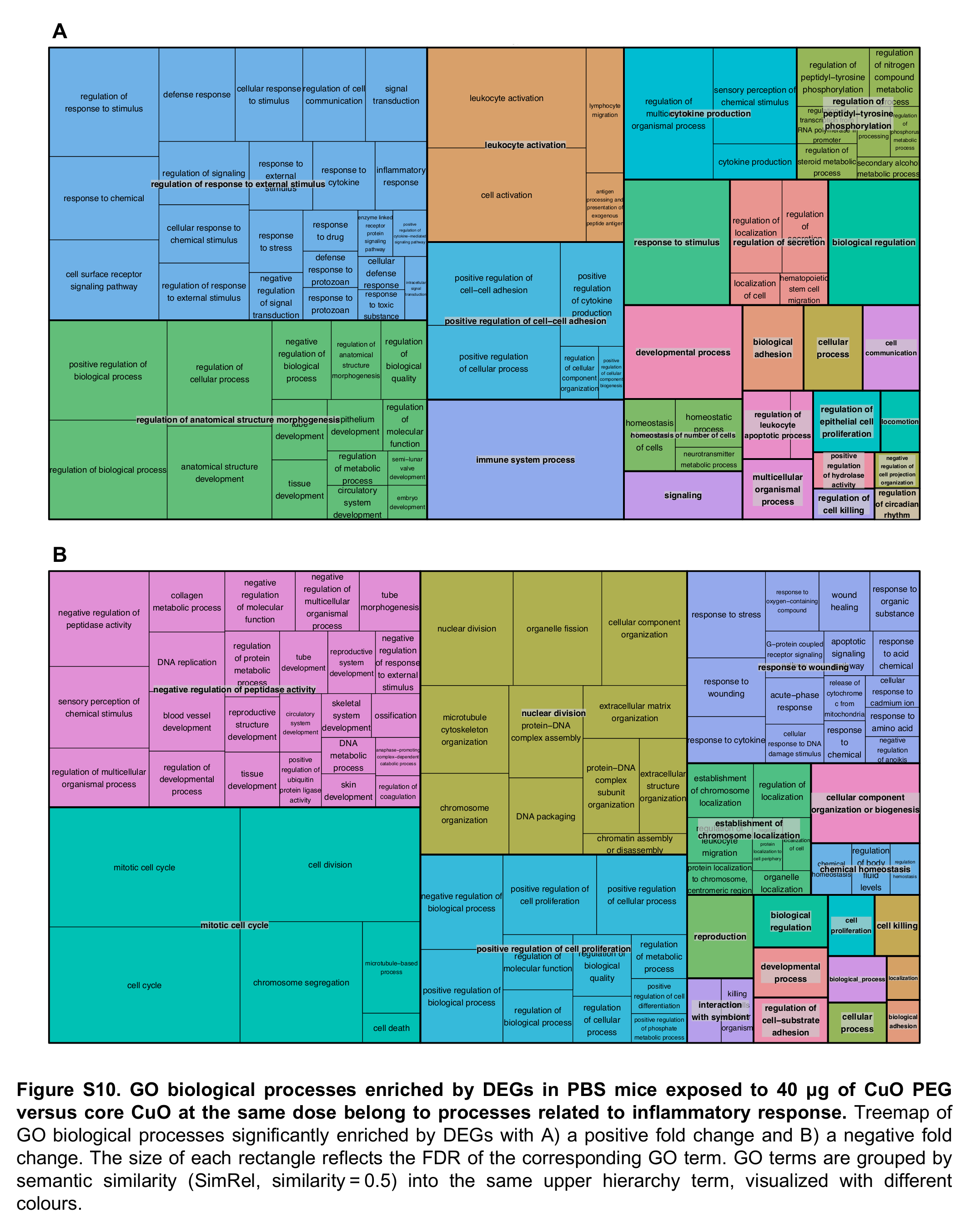

Supplement: Supplementary file 14 — Figure S10. GO biological processes enriched by DEGs in PBS mice exposed to 40 μg of CuO PEG versus core CuO at the same dose belong to processes related to inflammatory response. Treemap of GO biological processes significantly enriched by DEGs with A) a positive fold change and B) a negative fold change. The size of each rectangle reflects the FDR of the corresponding GO term. GO terms are grouped by semantic similarity (SimRel, similarity = 0.5) into the same upper hierarchy term, visualized with different colours. (TIF 1880 kb) [file 12989_2019_309_MOESM14_ESM.tif]

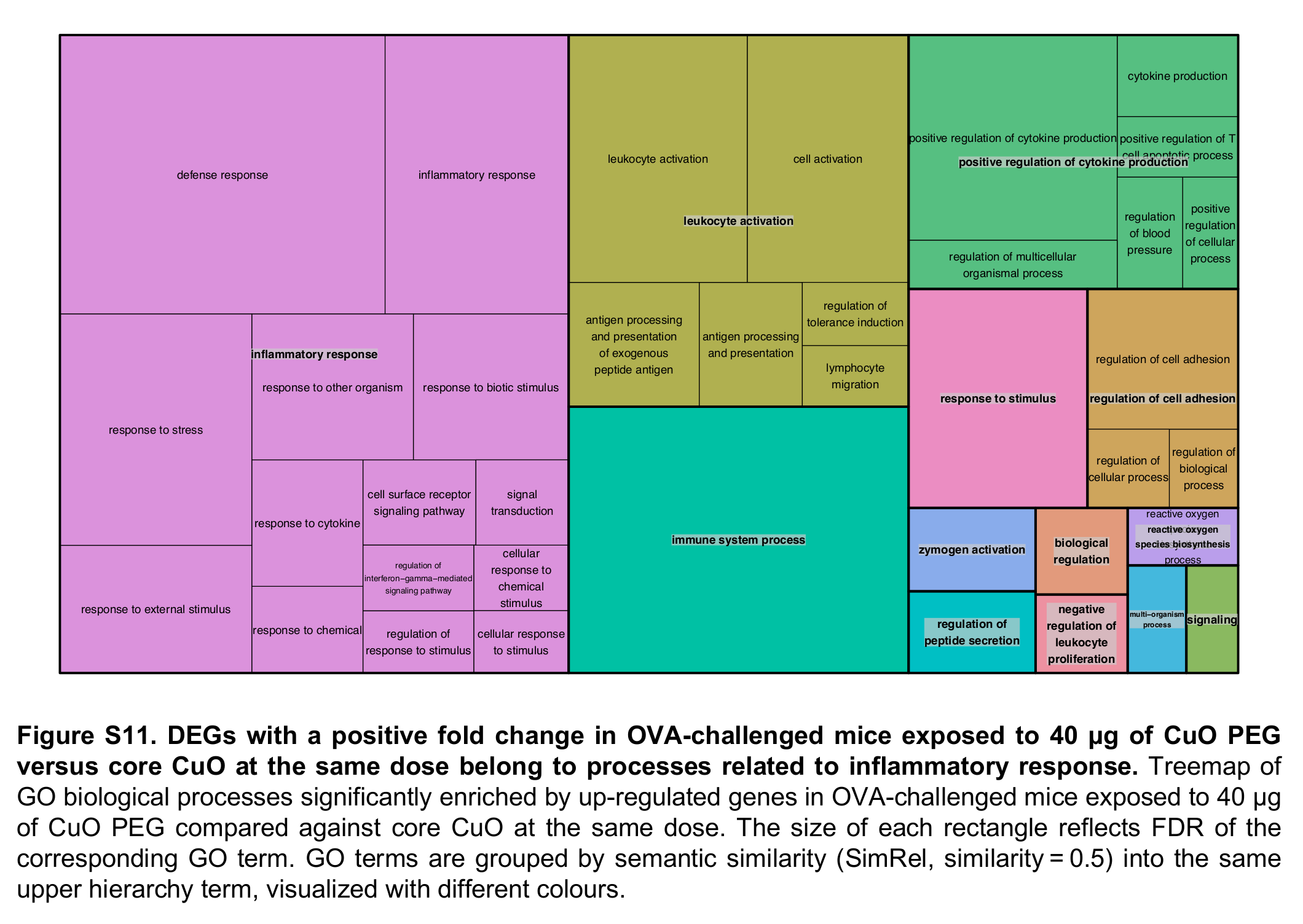

Supplement: Supplementary file 15 — Figure S11. DEGs with a positive fold change in OVA-challenged mice exposed to 40 μg of CuO PEG versus core CuO at the same dose belong to processes related to inflammatory response. Treemap of GO biological processes significantly enriched by up-regulated genes in OVA-challenged mice exposed to 40 μg of CuO PEG compared against core CuO at the same dose. The size of each rectangle reflects FDR of the corresponding GO term. GO terms are grouped by semantic similarity (SimRel, similarity = 0.5) into the same upper hierarchy term, visualized with different colours. (TIF 732 kb) [file 12989_2019_309_MOESM15_ESM.tif]

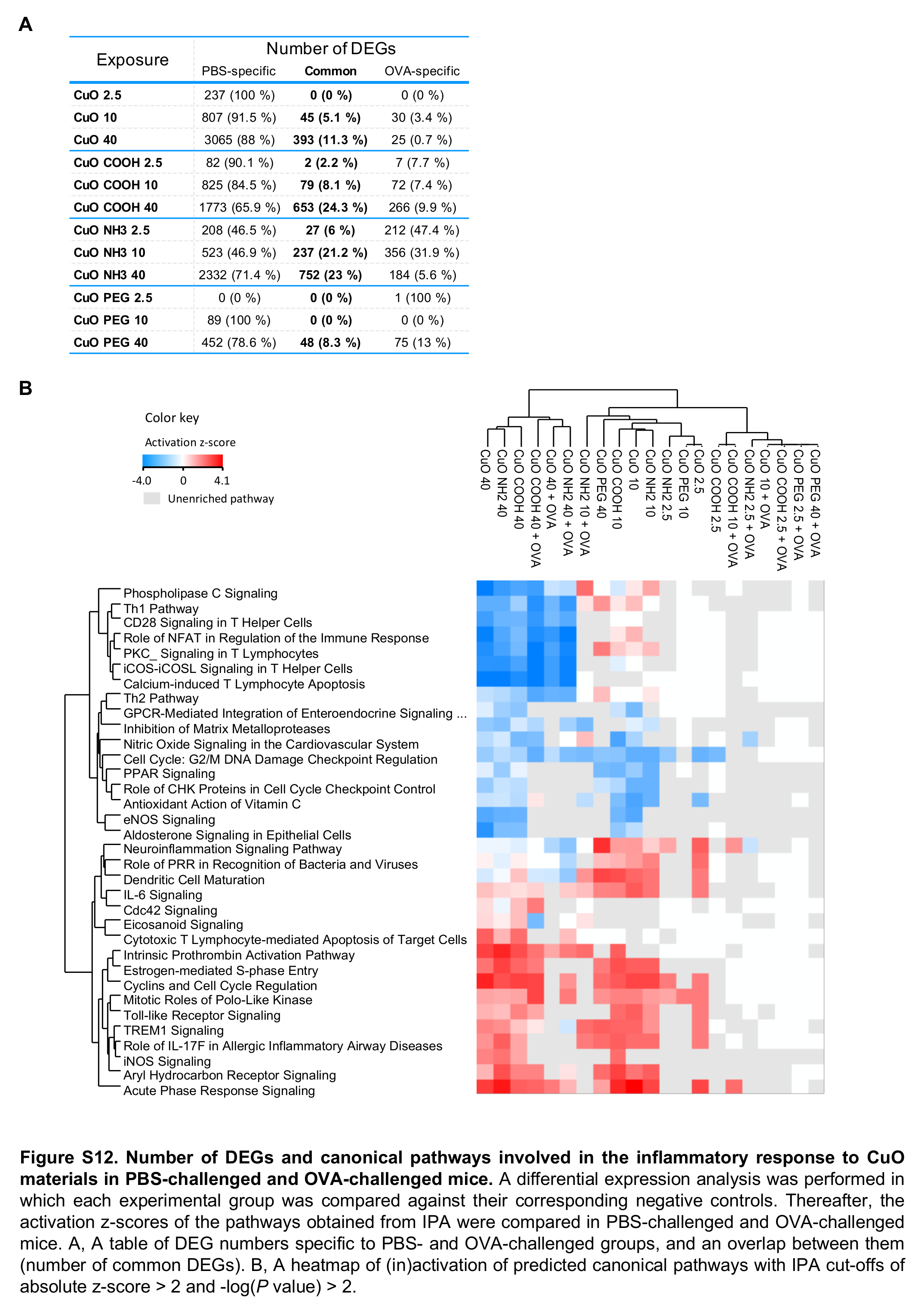

Supplement: Supplementary file 16 — Figure S12. Number of DEGs and canonical pathways involved in the inflammatory response to CuO materials in PBS-challenged and OVA-challenged mice. A differential expression analysis was performed in which each experimental group was compared against their corresponding negative controls. Thereafter, the activation z-scores of the pathways obtained from IPA were compared in PBS-challenged and OVA-challenged mice. A, A table of DEG numbers specific to PBS- and OVA-challenged groups, and an overlap between them (number of common DEGs). B, A heatmap of (in)activation of predicted canonical pathways with IPA cut-offs of absolute z-score > 2 and -log(P value) > 2. (TIF 1300 kb) [file 12989_2019_309_MOESM16_ESM.tif]

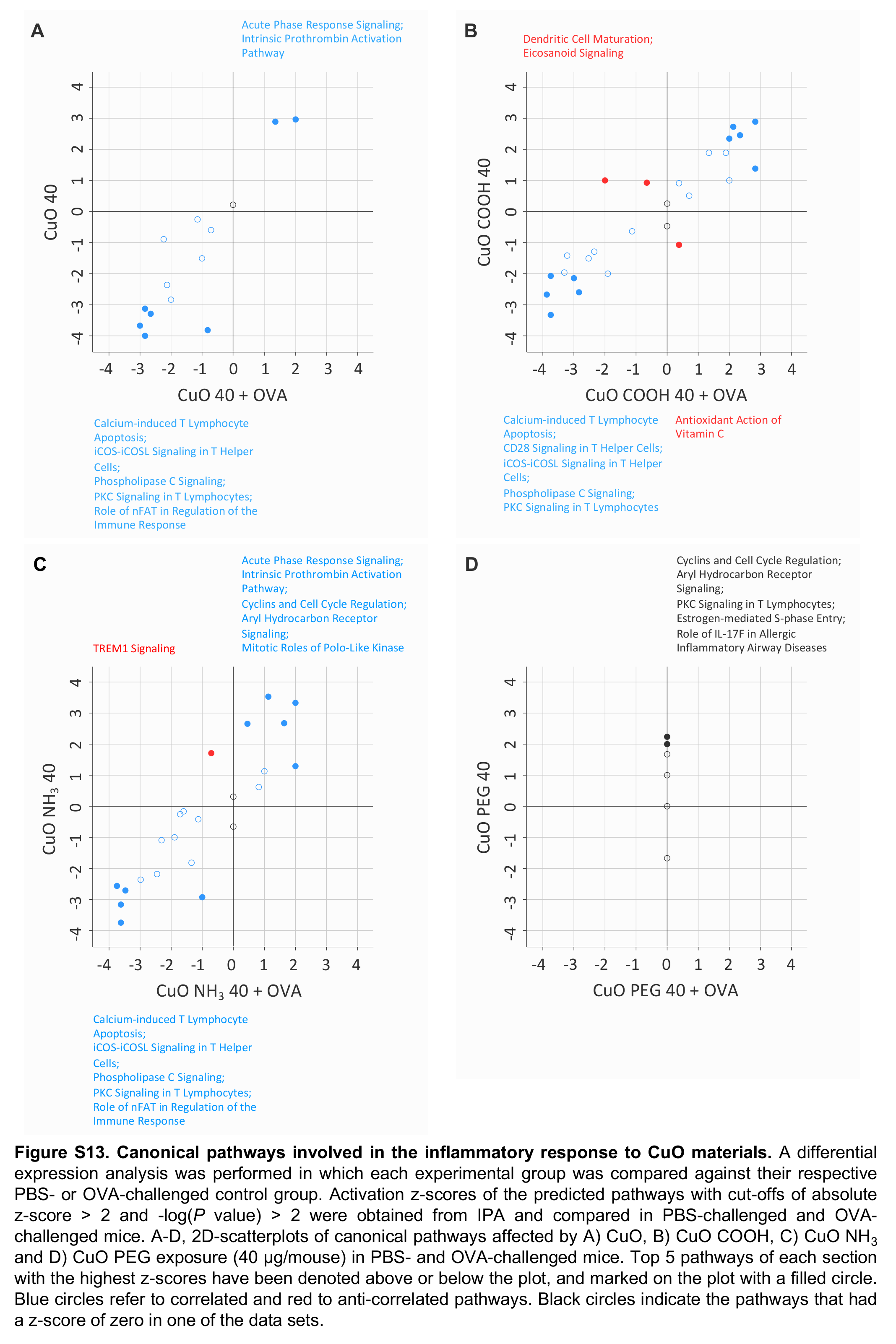

Supplement: Supplementary file 17 — Figure S13. Canonical pathways involved in the inflammatory response to CuO materials. A differential expression analysis was performed in which each experimental group was compared against their respective PBS- or OVA-challenged control group. Activation z-scores of the predicted pathways with cut-offs of absolute z-score > 2 and -log(P value) > 2 were obtained from IPA and compared in PBS-challenged and OVA-challenged mice. A-D, 2D-scatterplots of canonical pathways affected by A) CuO, B) CuO COOH, C) CuO NH3 and D) CuO PEG exposure (40 μg/mouse) in PBS- and OVA-challenged mice. Top 5 pathways of each section with the highest z-scores have been denoted above or below the plot, and marked on the plot with a filled circle. Blue circles refer to correlated and red to anti-correlated pathways. Black circles indicate the pathways that had a z-score of zero in one of the data sets. (TIF 1108 kb) [file 12989_2019_309_MOESM17_ESM.tif]
